# Exploring dynamic changes in metabolic profiles of *Artemisia argyi* leaves across different growth stages via plant metabolomics

**DOI:** 10.3389/fpls.2026.1913814

**Published:** 2026-08-11

**Authors:** Yue Zhong, Yiyang Ren, Jiayin Zhang, Jialei Liu, Yuguang Zheng, Dan Zhang, Lei Wang, Long Guo

**Affiliations:** 1 Traditional Chinese Medicine Processing Technology Innovation Center of Hebei Province, Hebei University of Chinese Medicine, Shijiazhuang, China; 2 International Joint Research Center on Resource Utilization and Quality Evaluation of Traditional Chinese Medicine of Hebei Province, Hebei University of Chinese Medicine, Shijiazhuang, China; 3 Hebei Chemical & Pharmaceutical College, Shijiazhuang, China; 4 Technology Innovation Center for Quantitative Research of Traditional Chinese Medicine, Hebei University of Chinese Medicine, Shijiazhuang, China

**Keywords:** *Artemisia argyi*, dynamic chemical profile, leaf development, mass spectrometry-based metabolomics, metabolism regulation

## Abstract

**Introduction:**

*Artemisia argyi* Levl. et Vant is a traditional Chinese medicinal herb with considerable medicinal value. *A. argyi* Levl. et Vant. leaves (AAL) have exhibited diverse biological activities that are tightly associated with their chemical components. During growth, the dynamic accumulation trends of bioactive metabolites were varied along with AAL developing. However, systematic studies on the dynamic variation patterns and regulatory mechanisms of major chemical constituents throughout their growth cycle remain scarce. The present study aims to clarify metabolic dynamic patterns of AAL during development.

**Methods:**

Mass spectrometry-based metabolomic platforms were applied for chemical profiling of AAL samples from 8 distinct growth stages. HS-GC-MS, pre-column derivatization GC-MS and UHPLC-Q-TOF-MS was applied for both volatile and non-volatile metabolite analysis. Orthogonal partial least squares-discriminant analysis and clustering analysis were further carried out for screening of differential components. A dynamic metabolism network of AAL was constructed by mapping annotated metabolites on their biosynthetic pathways.

**Results:**

HS-GC-MS enabled annotation of 44 volatile metabolites, comprising 36 monoterpenoids, 3 sesquiterpenoids, 3 alcohols, and 2 aromatic hydrocarbons. Pre-column derivatization GC-MS analysis allowed detection of 88 metabolites, including 38 sugar derivatives, 21 amino acids, 16 organic acids, and 13 miscellaneous metabolites. UHPLC-Q-TOF-MS further enabled the detection of additional of 44 metabolites, including 26 flavonoids, 12 phenolic acids, 5 coumarins, and 1 terpenoid. Most monoterpenoids and phenolic acids displayed significantly higher (*P* < 0.05) relative abundances at the early growth stage. Most flavonoids reached peak abundance at the vigorous growth stage. Sugar derivatives remained at low levels in AAL during the early growth stage, yet accumulated markedly at the vigorous and senescence stages. A proposed metabolic network encompassing pathways of carbohydrate, amino acid, flavonoid, terpenoid, and organic acid was constructed to further explore metabolism regulatory during AAL development.

**Discussion:**

The comprehensive profile of major metabolites in AAL across growth stages provides a scientific basis for the quality evaluation and sustainable exploitation of AAL medicinal resources.

## 1 Introduction

*Artemisia argyi* Lévl. et Vant is a perennial herb with high economic value, widely distributed across Asia and Europe (Liu et al., 2021). *A. argyi* leaves (“Aiye” in Chinese, AAL) are extensively utilized as pharmaceutical and aromatic ingredients in Asian countries (Miao et al., 2022). In traditional Chinese medicine (TCM), AAL are clinically employed for the treatment of inflammation, hemorrhage, pain, and dysmenorrhea (Xiao et al., 2019). Phytochemical investigations have demonstrated that AAL are rich in primary metabolites (e.g., amino acids, carbohydrates, dietary fibers) (Song et al., 2019; Yu et al., 2024) and secondary metabolites, including phenolic acids, flavonoids, essential oils, and terpenoids (Guo et al., 2023; Hu et al., 2024). These bioactive components endow AAL with diverse pharmacological activities, notably anti-inflammatory, antimicrobial, antioxidant, immunomodulatory, and microcirculation-improving effects. Besides clinical applications, AAL could also be processed into value-added products such as herbal teas, functional pastries, essential oils, and moxibustion formulations. Owing to their broad bioactivity spectrum and favorable safety profile, AAL hold substantial application potential in the pharmaceutical, functional food, cosmetic, and health maintenance sectors.

Harvest time across growth stages significantly affects the accumulation of chemical components in AAL. Young leaves picked around the Qingming solar term (early April) are preferred for making traditional foods (e.g., Qingtuan, a glutinous rice cake infused with AAL) due to their delicate texture and low contents of bitter compounds. Mature leaves harvested around the Dragon Boat Festival (the fifth day of the fifth month in the lunar calendar) exhibited peak levels of volatile oils and flavonoids (Xue et al., 2019; Chang et al., 2020), making them optimal raw materials for medicinal and moxibustion products. Seasonal variation of chemical profiles aligns with TCM harvesting wisdom. The Dragon Boat Festival, as a key agricultural solar term, not only carries cultural connotations of warding off evil spirits and preventing epidemics but also coincides with the natural rhythm of secondary metabolite accumulation in plants. Some existing studies have clarified the changes of total volatile oils, flavonoids, phenolic acids and several individual components of AAL during development (Xue et al., 2019; Chang et al., 2020). However, it is limited to reflect the harvest quality of AAL basing on these results. A more comprehensive profile reflecting the dynamic accumulation patterns of the overall chemical composition of AAL is necessary for determination of the optimal harvest time of AAL.

Plant metabolomics has emerged as a pivotal analytical technique with increasingly widespread applications in the quality evaluation of foods and traditional Chinese medicines. It enables systematic characterization of the dynamic changes in metabolites of AAL across different growth stages via targeted and untargeted approaches, thereby affording technical support for quality control (Yao et al., 2023). Ultra-high-performance liquid chromatography-quadrupole time-of-flight mass spectrometry (UHPLC-Q-TOF-MS) and gas chromatography-mass spectrometry (GC-MS) have become standard analytical platforms in plant metabolomics research owing to their high sensitivity and accuracy (Allwood and Goodacre, 2010; Feng et al., 2020). Among these techniques, headspace GC-MS (HS-GC-MS) permits direct detection of volatile components without elaborate sample pretreatment (Tholl et al., 2006), whereas pre-column derivatization GC-MS improves the detectability of non-volatile primary metabolites (Lisec et al., 2006). Nevertheless, GC-MS is unsuitable for analyzing secondary metabolites with poor thermal stability and low volatility, and this technical limitation can be effectively addressed by UHPLC-Q-TOF-MS (De Vos et al., 2007). The development of chemometric tools such as principal component analysis, orthogonal partial least squares-discriminant analysis (OPLS-DA), and clustering analysis has facilitated metabolomic data mining (Tan et al., 2021).

To elucidate the dynamic variation patterns and regulatory mechanisms of major chemical components in AAL throughout its growth cycle, HS-GC-MS, pre-column derivatization GC-MS, and UHPLC-Q/TOF-MS combined with multivariate data analysis were applied to systematically investigate metabolic variation across multiple growth stages. The data analysis results are anticipated to draw the dynamic chemical profile of growing AALs, which would provide a scientific foundation for the determination of the best harvest time. This would promote the transformation of AAL harvesting from empirical practice to precise and standardized cultivation. The systematic overview of the AAL chemical profile offers practical value for enhancing the utilization efficiency of Chinese medicinal material resources and advancing the high-quality development of the TCM industry.

## 2 Materials and methods

### 2.1 Plant materials

A total of 48 batches of *Artemisia argyi* Lévl. et Vant leaves (AAL), representing 8 distinct growth stages, were collected from the Medicinal Plant Garden of Hebei University of Chinese Medicine (Coordinates: 115°23′27.6″ E; 38°26′27.6″ N; Altitude: 25.15 m), Anguo, Hebei Province, China. At each harvesting time-point, six independent biological replicates were prepared. Each biological replicate consisted of pooled tissues collected from three randomly selected individual plants to reduce plant-to-plant variation. To ensure sampling consistency, leaves were collected simultaneously at the same height on multiple plants. All samples were identified by Professor Guo Long, and the collection dates of AAL samples at the 8 distinct growth stages are presented in **Supplementary Table 1** and **Supplementary Figure 1**. All specimens were preserved in the Herbarium of Hebei University of Chinese Medicine with their specimen numbers listed in **Supplementary Table 1**. Based on leaf developmental characteristics, the eight sampling stages were grouped into three phases which are early growth (S1 and S2), vigorous growth (S3-S5), and senescence (S6-S8) phases.

### 2.2 Chemicals and reagents

N-hydroxy pyridine (Purity ≥ 99.9%) and 2-isopropylmalic acid, were purchased from Aladdin Biochemical Technology Co., Ltd. (Shanghai, China). Methoxylamine hydrochloride (GC grade) and N-methyl-N-trimethylsilyl trifluoroacetamide (MSTFA, GC grade) were purchased from Sigma-Aldrich (St. Louis, MO, USA). Liquid chromatography-mass spectrometry (LC-MS) grade methanol, acetonitrile, and formic acid were purchased from Fisher Scientific (Pittsburgh, PA, USA). Ultrapure water was prepared using a Synergy water purification system (Millipore, Billerica, MA, USA). All other chemicals and reagents used were analytical grade.

### 2.3 Sample preparation and chemical analysis

#### 2.3.1 HS-GC–MS for volatile components analysis

For volatile component analysis, exactly 0.5 g of each sample powder was weighed into a 20 mL headspace vial and hermetically sealed immediately. The analysis was performed using an Agilent 7890B gas chromatograph equipped with a 7697A headspace sampler and an Agilent 5977B mass selective detector. An electron ionization (EI) source was used with the ionization energy set to 70 eV. Analytes were separated on an Agilent HP-5 MS capillary column (5% phenylmethyl polysiloxane, 0.25 mm × 30 m, 0.25 μm) with helium (99.999%) as the carrier gas at a flow rate of 1.1 mL/min. Headspace parameters were set as follows: sample oven temperature, 90 °C; loop temperature, 100 °C; transfer line temperature, 110 °C; equilibrium time, 15 min. The column temperature program was set as follows: initial temperature 60 °C (4 min), then increased to 90 °C at 5 °C/min (4 min), raised to 150 °C at 5 °C/min (2 min), and finally ramped to 200 °C at 12 °C/min (2 min). The temperatures of the ion source and quadrupole were 230 °C and 150 °C, respectively. A solvent delay of 3 min was applied, and detection was performed in full-scan mode (m/z 40–400).

#### 2.3.2 Pre-column derivatization GC-MS for analysis of non-volatile primary metabolites

AAL samples were ground to fine powder using a universal grinder and passed through a 40-mesh sieve, then dried to constant weight. Subsequently, 0.1 g of the dried AAL powder was accurately weighed and carefully transferred into a stoppered Erlenmeyer flask. A 1 mL of 75% methanol was then added as an extraction solution, and the mixture was ultrasonicated for 60 min with a power setting of 400 W. The mixture was then cooled to room temperature and reweighed to compensate for any weight loss. Afterwards, the mixture was centrifuged at 13,000 r·min^−1^ for 10 min. The resulting supernatant was collected and filtered through a 0.22 μm membrane (Agilent, Santa Clara, CA, USA). To ensure the stability of the analytical method, quality control (QC) samples were prepared by pooling equal aliquots of powder from all AAL samples.

For pre-column derivatization, 150 µL of the extract was mixed with 60 µL of 2-isopropylmalic acid (1 mg·mL^−1^, as the internal standard, IS) in a screw-cap vial, and evaporated to dryness under a nitrogen stream at 40 °C. Subsequently, 60 µL of methoxyamine hydrochloride (40 mg·mL^−1^ in pyridine) was added to the vial, followed by incubation in a metal bath at 30 °C for 90 min. For silylation, 240 µL of N-methyl-N-(trimethylsilyl)-trifluoroacetamide (MSTFA) was then added to the mixture, and incubation was continued at 37 °C for 30 min. Finally, the sample was centrifuged at 13,000 r·min^−1^ for 10 min, and the supernatant was collected for GC-MS analysis.

A 1 µL aliquot of the prepared supernatant was injected in split mode with a split ratio of 5:1, and the injection port temperature was set at 250 °C. The column temperature program was set as follows: initial temperature 80 °C, ramped to 200 °C at 12 °C·min^−1^, held for 1 min, then ramped to 250 °C at 8 °C·min^−1^, followed by a final ramp to 320 °C at 12 °C·min^−1^, held for 6 min. Ion energy was 70 eV. The interface temperature, ion source temperature, and quadrupole temperature were maintained at 250 °C, 230 °C, and 150 °C, respectively. Total ion chromatogram (TIC) scanning was performed over a mass range of m/z 50-500, with a solvent delay set at 2 min.

#### 2.3.3 UHPLC-Q/TOF-MS for analysis of non-volatile secondary metabolites

An accurately weighed 0.2 g of AAL powder was dissolved in 10 mL of 75% (v/v) methanol, following the same extraction procedure used for pre-column derivatization GC-MS analysis. A 1 µL of the above-mentioned AAL extract was injected into a UHPLC-Q-TOF/MS system consisting of an Agilent 1290 Infinity II system coupled with an Agilent 6545 Q-TOF-MS system (Agilent Technologies, Santa Clara, CA, USA), equipped with an electrospray ionization (ESI) source for secondary metabolite analysis. Chromatographic separation was performed on an Agilent ZORBAX SB-C18 column (4.6 × 50 mm, 1.8 μm, Santa Clara, CA, USA). A binary gradient elution system was employed, consisting of 0.1% formic acid in water (phase A) and acetonitrile (phase B). The separation was conducted at a flow rate of 0.4 mL·min^−1^, with the gradient program as follows: 0–6 min, 10% phase B; 6–11 min, phase B was increased from 10% to 16%; 11–23 min, phase B was increased from 16% to 23%; 23–28 min, phase B was increased from 23% to 26%; 28–38 min, phase B was increased from 26% to 31%; 38–45 min, 31% phase B; 45–48 min, phase B was increased from 31% to 43%; 48–53 min, phase B was increased from 43% to 54%. The column temperature was maintained at 25 °C.

Mass spectrometry acquisition parameters were set as follows: drying gas (N_2_) temperature at 320 °C; sheath gas (N_2_) temperature at 350 °C; drying gas (N_2_) flow rate at 10.0 L·min^−1^; sheath gas (N_2_) flow rate at 11 L·min^−1^; nebulizer gas (N_2_) pressure at 35 psi; capillary voltage at 3500 V; fragmentor voltage at 135 V; collision energy at 40 eV. Analysis was carried out in positive-ion mode, with a mass-to-charge ratio (m/z) range of 100-1000.

### 2.4 Data preprocessing and compound annotation

After mass spectrometric detection, raw files obtained from GC-MS analysis were processed with Agilent MassHunter Workstation Qualitative Analysis software. For data deconvolution, the retention time window scaling factor was set to 100 to integrate extracted ion chromatograms (EICs) into compound features. The ion at m/z 28 was excluded to eliminate nitrogen interference, and ions with absolute peak heights below 500 counts and absolute peak areas below 1000 counts were filtered out. All remaining parameters were kept as default values, and compound annotation was further performed by searching against the NIST 17 database. For data obtained from pre-column derivatization analysis, the peak area of each annotated metabolite was normalized to that of internal standard 2-isopropylmalic acid. The resulting normalized peak-area profiles were used to characterize stage-dependent changes of the annotated primary metabolites.

Raw files from UHPLC-Q/TOF-MS analysis were processed via Agilent MassHunter Qualitative Analysis Software (Version B.10.00, Agilent Technologies, Santa Clara, CA, USA) and Profinder software. Annotation of non-volatile compounds was conducted by searching against databases such as MassBank (https://massbank.eu/MassBank/Search), Agilent Herbal Library-v20–04–17, ChemSpider (http://www.chemspider.com/), PubChem (https://pubchem.ncbi.nlm.nih.gov/), and relevant literature. The peak areas of annotated metabolites were normalized to that of internal standard psoralen, and the resulting normalized peak-area profiles were used to characterize stage-dependent changes in the relative abundance of secondary metabolites.

### 2.5 Screening of differential components and statistical analysis

Peak areas of annotated metabolites were extracted, normalized to internal standards, and then imported into SIMCA software (Version 14.0, Umetrics, Umeå, Sweden) for orthogonal partial least squares-discriminant analysis (OPLS-DA).

Statistical significance across growth stages was assessed by one-way analysis of variance (ANOVA) followed by Duncan’s multiple-range test, with *P*<0.05 considered significant. Subsequently, differential chemical components of AAL were screened based on the variable importance in projection (VIP) values (VIP > 1) derived from the OPLS-DA model. Cluster analysis (CA) was performed using OriginPro 2021 (Version 9.8, OriginLab Corporation, Northampton, MA, USA).

### 2.6 KEGG pathway analysis of the annotated metabolites

Annotated metabolites were mapped to metabolic pathways via the MetaboAnalyst 6.0 platform (https://www.metaboanalyst.ca/home.xhtml) using annotations from the Kyoto Encyclopedia of Genes and Genomes (KEGG). Metabolite set enrichment analysis (MSEA) prioritized metabolic pathways by *P*-value (y-axis, pathway enrichment analysis) or pathway impact (x-axis, pathway topology analysis). Significance was determined via a hypergeometric test with an adjusted *P* < 0.05 cutoff.

## 3 Results and discussion

An integrated mass spectrometry-based metabolomics strategy, incorporating HS-GC-MS) pre-column derivatization-GC-MS, and UHPLC-Q/TOF-MS, alongside multivariate statistical analysis, was employed to systematically characterize the dynamic variation profiles of chemical constituents in AAL across different growth stages. Comprehensive analysis of dynamic chemical patterns of AAL would provide experimental support for optimal harvest-time selection according to various purpose.

### 3.1 Metabolite detection and annotation

#### 3.1.1 Detection of volatile components by HS-GC-MS

**Figure 1:**
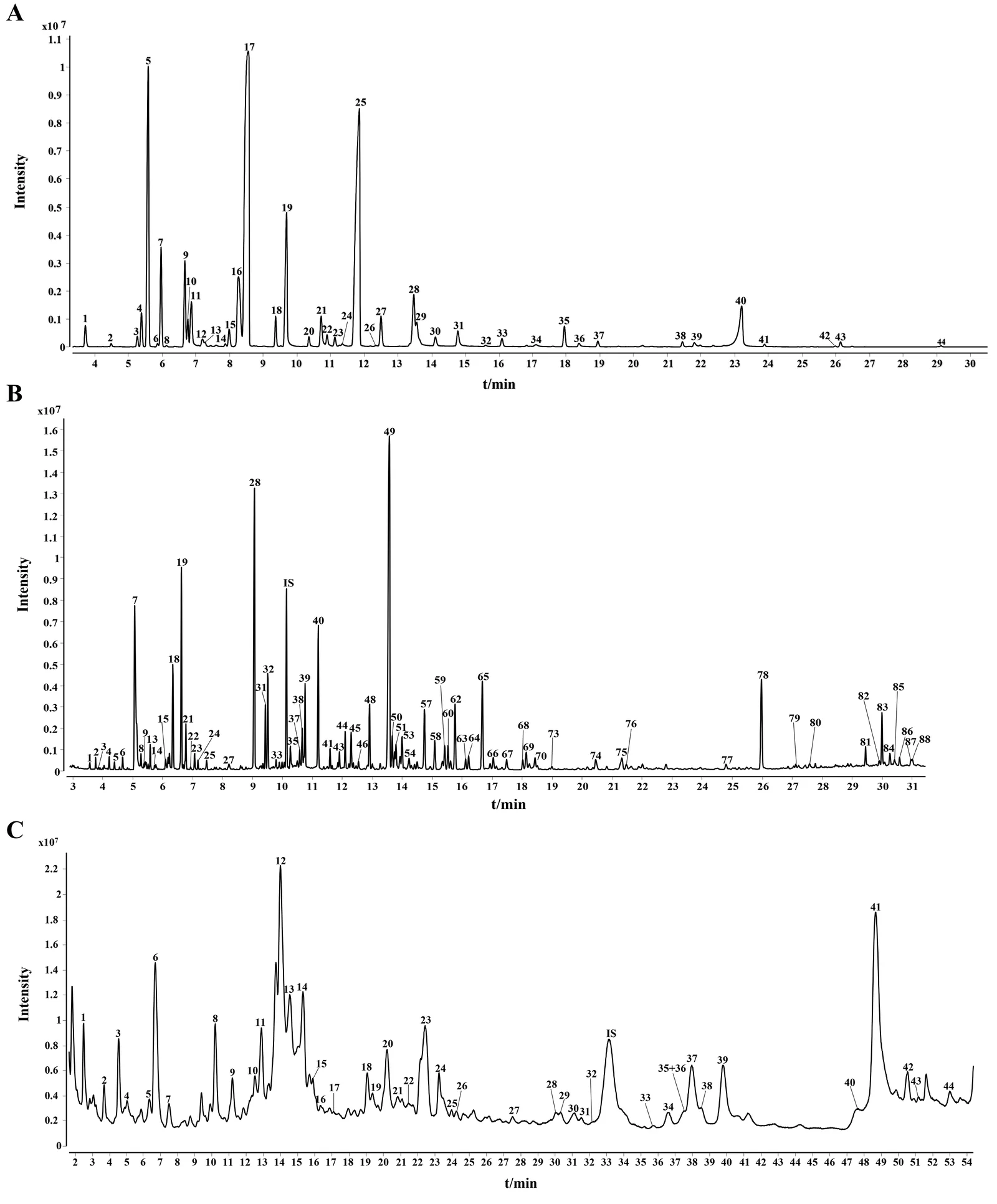
Total ion chromatograms (TICs) of metabolites in AAL. **(A)** Volatile components analyzed by HS-GC-MS; **(B)** primary metabolites analyzed by pre-column derivatization GC-MS; **(C)** secondary metabolites analyzed by UHPLC-Q/TOF-MS in positive electrospray ionization mode.

HS-GC-MS was employed to detect volatile metabolites in AAL across different growth stages. The typical total ion chromatogram (TIC) of GC-MS is shown in **Figure 1A**. By searching against the National Institute of Standards and Technology (NIST 17) library, a total of 44 volatile metabolites were annotated (**Table 1**), including 36 monoterpenes, 3 sesquiterpenes, 3 alcohols, and 2 aromatic hydrocarbons.

**Table 1:** Information of annotated volatile components in *A. argyi* leaves.

No.	RT (min)	Molecular formula	Theoretical	Annotated compound	Classification	Annotation level
			mass (*m/z*)			
V1	3.77	C_9_H_16_O	140.2	3,6-Dimethyl-1-heptyne-3-ol*	Alcohols	2
V2	4.49	C_9_H_20_O	144.2	1-Nonanol	Alcohols	2
V3	5.29	C_10_H_16_	136.2	3-Carene	Monoterpenes	2
V4	5.40	C_10_H_16_	136.2	α-Pinene	Monoterpenes	2
V5	5.60	C_10_H_16_	136.2	Cyclofenchene*	Monoterpenes	2
V6	5.86	C_10_H_14_	134.2	1-Isopropyl-4-methylenebicyclo[3.1.0]hex-2-ene*	Monoterpenes	2
V7	5.97	C_10_H_16_	136.2	Camphene*	Monoterpenes	2
V8	6.13	C_10_H_14_	134.2	2,4-Thujadiene	Monoterpenes	2
V9	6.67	C_10_H_16_	136.2	Sabinene	Monoterpenes	2
V10	6.77	C_10_H_16_	136.2	β-Pinene	Monoterpenes	2
V11	6.88	C_8_H_16_O	128.2	1-Octen-3-ol*	Alcohols	2
V12	7.20	C_10_H_16_O	152.2	1,3,3-Trimethyl-2-oxabicyclo[2.2.2]oct-5-ene*	Monoterpenes	2
V13	7.28	C_9_H_12_	120.1	1,2,4-Trimethylbenzene*	Aromatic hydrocarbons	2
V14	7.89	C_10_H_12_	132.2	2-Methyl-1-phenylpropene*	Aromatic hydrocarbons	2
V15	7.99	C_10_H_16_	136.2	α-Terpinene*	Monoterpenes	2
V16	8.27	C_10_H_14_	134.2	p-Cymene*	Monoterpenes	2
V17	8.51	C_10_H_18_O	154.2	1,8-Cineole*	Monoterpenes	2
V18	9.40	C_10_H_16_	136.2	γ-Terpinene*	Monoterpenes	2
V19	9.69	C_10_H_18_O	154.2	Sabinene hydrate	Monoterpenes	2
V20	10.34	C_10_H_18_O	154.2	3,3,6-Trimethylhepta-1,5-dien-4-ol*	Monoterpenes	2
V21	10.71	C_10_H_18_O	154.2	4-Thujanol	Monoterpenes	2
V22	10.93	C_10_H_16_O	152.2	(-)-α-Thujone	Monoterpenes	2
V23	11.14	C_10_H_14_O	150.2	(+)-Chrysanthenone*	Monoterpenes	2
V24	11.36	C_10_H_16_O	152.2	cis-Chrysanthenol	Monoterpenes	2
V25	11.78	C_10_H_14_O	150.2	Eucarvone	Monoterpenes	2
V26	12.35	C_10_H_16_O	152.2	(-)-trans-Pinocarveol	Monoterpenes	2
V27	12.49	C_10_H_16_O	152.2	Camphor*	Monoterpenes	2
V28	13.49	C_10_H_16_O	152.2	Trans-Chrysanthenol	Monoterpenes	2
V29	13.55	C_10_H_18_O	154.2	Borneol*	Monoterpenes	2
V30	14.14	C_10_H_18_O	154.2	Terpinen-4-ol	Monoterpenes	2
V31	14.80	C_10_H_18_O	154.2	α-Terpineol	Monoterpenes	2
V32	15.62	C_10_H_14_O	150.2	(-)-Verbenone	Monoterpenes	2
V33	16.10	C_10_H_18_O	154.2	γ-Terpineol	Monoterpenes	2
V34	17.09	C_10_H_14_O	150.2	2,6-Dimethylocta-2,5,7-trien-4-one	Monoterpenes	2
V35	17.96	C_10_H_16_O	152.2	(+)-cis-Verbenol	Monoterpenes	2
V36	18.39	C_10_H_14_O	150.2	(+)-Isopiperitenone	Monoterpenes	2
V37	18.93	C_12_H_20_O_2_	196.2	L-Bornyl acetate	Monoterpenes	2
V38	21.47	C_12_H_20_O_2_	196.2	α-Terpinyl acetate	Monoterpenes	2
V39	21.78	C_10_H_12_O_2_	164.2	Eugenol	Monoterpenes	2
V40	23.15	C_10_H_14_O	150.2	3-Caren-5-one	Monoterpenes	2
V41	23.87	C_15_H_24_	204.3	Caryophyllene	Monoterpenes	2
V42	25.97	C_15_H_24_	204.3	Germacrene D	Sesquiterpenes	2
V43	26.14	C_15_H_24_	204.3	β-Selinene*	Sesquiterpenes	2
V44	29.141	C_15_H_24_O	220.3	Caryophyllene oxide	Sesquiterpenes	2

*VIP > 1.

Annotation confidence levels were assigned according to Sumner et al. (2007). Level 1: confirmed identification using an authentic standard and at least two independent and orthogonal properties obtained under the same experimental conditions. Level 2: putatively annotated compound based on spectral-library, accurate-mass, fragment-ion, database, and/or literature matching without confirmation using an authentic standard. Level 3: putatively characterized compound class based on class-specific spectral or physicochemical properties. Level 4: unknown or unclassified compound.

#### 3.1.2 Detection of non-volatile primary metabolites by pre-column derivatization-GC-MS

For non-volatile polar metabolites, pre-column derivatization was implemented to increase their volatility and thermal stability before analysis with GC-MS. **Figure 1B** shows the typical TIC of derivatized AAL extracts analyzed by GC-MS. A total of 88 metabolites were annotated (**Figure 1B** and **Table 2**), including 38 carbohydrate derivatives, 21 amino acids, 16 organic acids, and 13 other metabolites. These compounds are key metabolites involved in primary metabolism.

**Table 2:** Information of annotated primary metabolites in *A. argyi* leaves.

No.	RT (min)	Molecular	Theoretical	Annotated metabolite	Classification	Annotation level
P1	3.76	C_9_ H_22_ O_3_ Si_2_	234.4	Lactic Acid, 2TMS*	Organic acid	2
P2	3.86	C_9_H_20_O_2_Si	188.3	Hexanoic acid, TMS	Fatty Acids	2
P3	3.90	C_8_H_20_O_3_Si_2_	220.4	Glycolic acid, 2TMS	Organic acid	2
P4	4.22	C_9_H_23_NO_2_Si_2_	233.4	L-Alanine, 2TMS*	Amino acids	2
P5	4.39	C_9_H_27_NOSi_3_	249.5	Hydroxylamine, 3TMS	Nitrogenous substances	2
P6	4.66	C_8_H_18_O_4_Si_2_	234.4	Oxalic acid, 2TMS*	Organic acid	2
P7	5.09	C_8_H_17_NO_2_Si_2_	187.3	L-Proline, TMS	Amino acids	2
P8	5.28	C_9_H_18_O_2_Si	186.1	Cyclopentylcarboxylic acid, TMS	Organic acid	2
P9	5.38	C_13_H_24_OSi	224.2	(-)-Myrtenol, TMS	Terpenoids	2
P10	5.41	C_9_H_20_O_4_Si_2_	248.4	Propanedioic acid, 2TMS	Organic acid	2
P11	5.45	C_13_H_26_OSi	226.2	Neryl alcohol, TMS	Terpenoids	2
P12	5.52	C_12_H_29_NO_2_Si_2_	275	L-Leucine, 2TMS*	Amino acids	2
P13	5.60	C_11_H_27_NO_2_Si_2_	261.5	L-Valine, 2TMS*	Amino acids	2
P14	5.73	C_13_H_26_OSi	226.2	Isopulegol, 2TMS	Alcohols	2
P15	6.10	C_9_H_23_NO_3_Si_2_	249.4	L-Serine, 2TMS*	Amino acids	2
P16	6.18	C_9_H_19_NO_2_Si	201.3	Pipecolic acid, TMS	Amino acids	2
P17	6.22	C_11_H_31_NOSi_3_	277.6	Ethanolamine, 3TMS*	Nitrogenous substances	2
P18	6.34	C_12_H_32_O_3_Si_3_	308.6	Glycerol, 3TMS	Carbohydrates	2
P19	6.63	C_14_H_36_N_2_O_2_Si_3_	348.2	L-Ornithine, 3TMS	Amino acids	2
P20	6.69	C_10_H_20_O_4_Si_2_	260.1	Maleic Acid, 2TMS*	Organic acid	2
P21	6.77	C_10_H_22_O_4_Si_2_	262.4	Succinic Acid, 2TMS	Organic acid	2
P22	6.93	C_18_H_34_OSi	294.2	β-Eudesmol, TMS	Terpenoids	2
P23	7.07	C_12_H_30_O_4_Si_3_	322.6	Glyceric acid, 3TMS	Organic acid	2
P24	7.17	C_10_H_24_N_2_O_2_Si_2_	260.1	Acetic Acid, 2TMS	Organic acid	2
P25	7.46	C_12_H_27_NO_2_Si_2_	273.5	Cycloleucin, 2TMS*	Amino acids	2
P26	7.80	C_13_H_33_NO_3_Si_3_	335	L-Threonine, 3TMS	Amino acids	2
P27	8.21	C_11_H_29_NO_2_Si_3_	291	Glycine, 3TMS	Amino acids	2
P28	9.05	C_13_H_30_O_5_Si_3_	350.6	Malic acid, 3TMS*	Organic acid	2
P29	9.28	C_13_H_22_O_3_Si_2_	282.1	Salicylic acid, 2TMS	Organic acid	2
P30	9.34	C_16_H_42_O_4_Si_4_	410.2	L-Threitol, 4TMS*	Sugar alcohols	2
P31	9.43	C_13_H_31_NO_4_Si_3_	349.6	L-Aspartic acid, 3TMS	Amino acids	2
P32	9.51	C_13_H_33_NO_2_Si_3_	319.6	4-Aminobutyric acid, 3TMS	Amino acids	2
P33	9.78	C_16_H_41_NO_4_Si_4_	423.2	L-Threose, 4TMS*	Carbohydrates	2
P34	9.89	C_21_H_53_NO_5_Si_5_	539.3	L-(-)-Fucose, 5TMS*	Carbohydrates	2
IS	10.14	C_16_H_36_O_5_Si_3_	392.2	2-Isopropylmalic acid, 3TMS	Fatty Acids	2
P35	10.26	C_17_H_42_NO_5_Si_4_	452.2	Xylose, 4TMS	Carbohydrates	2
P36	10.49	C_12_H_30_N_2_O_2_Si_3_	318.2	Cycloserine, 3TMS*	Amino acids	2
P37	10.57	C_14_H_33_NO_4_Si_3_	363.6	L-Glutamic acid, 3TMS*	Amino acids	2
P38	10.64	C_15_H_27_NO_2_Si_2_	309.5	Phenylalanine, 2TMS*	Amino acids	2
P39	10.76	C_13_H_32_N_2_O_3_Si_3_	348.2	L-Asparagine, 3TMS*	Amino acids	2
P40	11.18	C_13_H_32_N_2_O_3_Si_3_	348.2	Homoserine, 3TMS	Amino acids	2
P41	11.58	C_20_H_52_O_5_Si_5_	512.3	D-Arabitol, 5TMS	Sugar alcohols	2
P42	11.84	C_20_H_52_O_5_Si_5_	512.3	Xylitol, 5TMS*	Sugar alcohols	2
P43	11.90	C_18_H_46_N_2_O_2_Si_4_	434.3	Lysine, 4TMS*	Amino acids	2
P44	12.08	C_16_H_42_O_4_Si_4_	410.2	Erythritol, 4TMS*	Carbohydrates	2
P45	12.28	C_14_H_34_N_2_O_3_Si_3_	362.2	L-Glutamine, 3TMS*	Amino acids	2
P46	12.53	C_21_H_52_O_6_Si_5_	540.3	Psicofuranose, 4TMS	Carbohydrates	2
P47	12.89	C_18_H_40_O_7_Si_4_	480.2	Citric acid, 4TMS	Organic acid	2
P48	12.98	C_19_H_46_O_6_Si_4_	482.2	Methyl galactoside, 4TMS	Carbohydrates	2
P49	13.55	C_22_H_54_O_6_Si_5_	554.3	D-Pinitol, 5TMS	Sugar alcohols	2
P50	13.66	C_22_H_55_NO_6_Si_5_	569.3	D-Fructose, 5TMS	Carbohydrates	2
P51	13.74	C_22_H_55_NO_6_Si_5_	569.3	D-Galactose, 5TMS*	Carbohydrates	2
P52	13.78	C_22_H_55_NO_6_Si_5_	569.3	L-(-)-Sorbose, 5TMS	Carbohydrates	2
P53	13.98	C_22_H_55_NO_6_Si_5_	569.3	D-Allose, 5TMS	Carbohydrates	2
P54	14.30	C_18_H_35_NO_3_Si7	397.2	L-Tyrosine, 3TMS*	Amino acids	2
P55	14.39	C_24_H_62_O_6_Si_6_	615.3	L-Sorbitol, 6TMS*	Sugar alcohols	2
P56	14.49	C_12_H_28_O_5_Si_3_	336.1	Tartronic acid, 3TMS	Organic acid	2
P57	14.73	C_21_H_52_O_6_Si_5_	540.3	β-D-Galactofuranose, 5TMS	Carbohydrates	2
P58	15.07	C_24_H_60_O_6_Si_6_	612.3	D-Tagatose, 6TMS	Carbohydrates	2
P59	15.41	C_24_H_60_O_7_Si_6_	628.3	D-Gluconic acid, 6TMS*	Carbohydrates	2
P60	15.50	C_19_H_40_O_2_Si	328.3	Palmitic acid, TMS	Fatty Acids	2
P61	15.60	C_24_H_58_O_8_Si_6_	642.3	Galactaric acid, 6TMS	Fatty Acids	2
P62	15.75	C_21_H_52_O_6_Si_5_	540.3	D-Allofuranose, 5TMS*	Carbohydrates	2
P63	16.10	C_17_H_42_O_5_Si_4_	438.2	D-Ribofuranose, 4TMS*	Carbohydrates	2
P64	16.20	C_18_H_44_O_5_Si_4_	452.2	Deoxyglucose, 4TMS	Carbohydrates	3
P65	16.66	C_24_H_60_O_6_Si_6_	612.3	Myo-Inositol, 6TMS*	Carbohydrates	2
P66	17.02	C_18_H_32_O_4_Si_3_	396.2	Caffeic acid, 3TMS	Organic acid	2
P67	17.48	C_23_H_48_OSi	368.3	Phytol, TMS*	Terpenoids	2
P68	18.02	C_21_H_40_O_2_Si	352.6	Linoleic acid, TMS	Fatty Acids	2
P69	18.13	C_21_H_38_O_2_Si	350.6	α-Linolenic acid, TMS	Fatty Acids	2
P70	18.24	C_18_H_44_O_5_Si_4_	452.2	L-Rhamnose, 4TMS*	Carbohydrates	2
P71	18.42	C_20_H_36_N_2_O_2_Si_3_	420.2	L-Tryptophan, 3TMS*	Amino acids	2
P72	18.49	C_21_H_44_O_2_Si	356	Stearic acid, TMS*	Fatty Acids	2
P73	18.98	C_20_H_48_O_6_Si_4_	496.3	α-D-Glucopyranoside, 4TMS*	Carbohydrates	3
P74	20.44	C_27_H_66_O_8_Si_6_	686.3	Glyceryl-glycoside, 6TMS*	Carbohydrates	3
P75	21.30	C_24_H_62_O_6_Si_6_	614.3	Galactitol, 6TMS	Sugar alcohols	2
P76	21.47	C_21_H_50_O_7_Si_5_	554	D-Galacturonic acid, 5TMS*	Carbohydrates	2
P77	24.79	C_36_H_86_O_11_Si_8_	918	D-Cellobiose, 8TMS	Carbohydrates	2
P78	25.97	C_36_H_86_O_11_Si_8_	918	Sucrose, 8TMS	Carbohydrates	2
P79	27.12	C_36_H_86_O_11_Si_8_	918	Maltose, 8TMS	Carbohydrates	2
P80	27.59	C_36_H_86_O_11_Si_8_	918	D-Lactose, 8TMS	Carbohydrates	2
P81	29.45	C_36_H_86_O_11_Si_8_	918	Melibiose, 8TMS	Carbohydrates	2
P82	29.99	C_34_H_66_O_9_Si_6_	786.3	Chlorogenic Acid, 6TMS*	Organic acid	2
P83	29.99	C_22_H_52_O_6_Si_5_	552.3	Quininic acid, 6TMS	Organic acid	2
P84	30.26	C_21_H_52_O_6_Si_5_	552.3	D-Glucopyranose, 5TMS	Carbohydrates	2
P85	30.42	C_34_H_66_O_9_Si_6_	786.3	D-(-)-Arabinose, 6TMS	Carbohydrates	2
P86	30.59	C_24_H_44_F_5_NO_5_Si_4_	633.2	Pentofuranose, 4TMS	Carbohydrates	3
P87	30.96	C_18_H_44_O_5_Si_4_	452.2	1,5-Anhydroglucitol, 4TMS	Sugar alcohols	2
P88	31.01	C_19_H_44_O_6_Si_4_	480.2	Sedoheptulose, 4TMS*	Carbohydrates	2

*VIP > 1.

Annotation confidence levels were assigned according to Sumner et al. (2007). Level 1: confirmed identification using an authentic standard and at least two independent and orthogonal properties obtained under the same experimental conditions. Level 2: putatively annotated compound based on spectral-library, accurate-mass, fragment-ion, database, and/or literature matching without confirmation using an authentic standard. Level 3: putatively characterized compound class based on class-specific spectral or physicochemical properties. Level 4: unknown or unclassified compound.

#### 3.1.3 Detection of non-volatile secondary metabolites by UHPLC-Q-TOF-MS

AAL contains diverse secondary metabolites, with phenolic acids and flavonoids as the major classes. To comprehensively characterize secondary metabolites in AAL, UHPLC-Q-TOF-MS was applied for metabolite annotation and relative-abundance profiling. Since most secondary metabolites in AAL exhibit prominent protonated molecular ions ([M+H]^+^), samples were analyzed in positive-ion mode. **Figure 1C** shows a typical TIC of AAL leaves analyzed by UHPLC-Q/TOF-MS. Based on fragmentation patterns (Zhu et al., 2015; Palmer-Young et al., 2016; Sisó-Terraza et al., 2016; Zhang et al., 2017; Gifford et al., 2018; Jiao et al., 2018; Lee et al., 2018; Fu et al., 2020; Hu et al., 2020; Attallah et al., 2021; Chang et al., 2021; Cui et al., 2021; Haile et al., 2021; Hernández et al., 2021; Luo et al., 2021; Palșca et al., 2021; Serrano et al., 2021; Basit et al., 2022; Gao et al., 2022; Li et al., 2022; Martín-García et al., 2022; Nasseri et al., 2022; Slimestad et al., 2022; Zhang et al., 2022; Bojilov et al., 2023; Darnal et al., 2023; El-Fadaly et al., 2023; El-Newary et al., 2023; Kamel et al., 2023; Ramos et al., 2023; Yu et al., 2023; Zhang et al., 2023; Zhang et al., 2024; Ali et al., 2025; Rodrigues et al., 2025; Wójcik-Borowska et al., 2025), a total of 44 secondary metabolites were annotated with characteristic molecular and fragment ions summarized in **Table 3**. The annotated metabolites included 12 phenolic acids, 26 flavonoids, 5 coumarins, and 1 terpenoid.

**Table 3:** Information of annotated secondary metabolites in *A. argyi* leaves.

No.	RT (min)	Molecular Formula	Molecular ion	Fragment ions (m/z)	Error	Annotation	Classification	Ref.	Annotation level
			(*m/z*)		(ppm)				
S1	2.45	C_16_H_18_O_9_	355.1037	181.0491、163.0395、135.0443	-1.96	5-Caffeoylquinic acid*	Phenolic acid	(Yu et al., 2023)	2
S2	3.70	C_15_H_16_O_9_	341.0868	179.0338、163.0390	-0.52	Esculin*	Coumarins	(Martín-García et al., 2022)	2
S3	4.55	C_16_H_18_O_10_	371.0971	209.1911、208.8147	-0.49	Fraxin*	Coumarins	(Sisó-Terraza et al., 2016)	2
S4	4.91	C_9_H_6_O_4_	179.0344	135.0444、123.0438	-1.26	Esculetin	Coumarins	(Attallah et al., 2021)	2
S5	5.88	C_15_H_16_O_8_	324.2021	163.0392、135.0418	0.97	Skimmin	Coumarins	(Zhang et al., 2017)	2
S6	6.67	C_16_H_18_O_9_	355.1037	183.1017、181.0492、163.0393、135.0444	0.53	3-Caffeoylquinic acid	Phenolic acid	(Yu et al., 2023)	2
S7	7.48	C_16_H_18_O_9_	355.1031	173.0433、163.0394、137.0579、135.0445、107.0478	-0.07	4-Caffeoylquinic acid*	Phenolic acid	(Palmer-Young et al., 2016; Yu et al., 2023)	2
S8	10.17	C_9_H_8_O_4_	181.0496	179.0360、163.0393、135.0432	-2.1	Caffeic acid	Phenolic acid	(Serrano et al., 2021)	2
S9	11.17	C_15_H_12_O_5_	273.0751	153.1253、151.0053、147.0444	2.11	Naringenin	Flavonoids	(Zhang et al., 2022)	2
S10	12.52	C_17_H_20_O_9_	369.1171	191.1026、118.0817	-0.89	5-Feruoyl quinic acid*	Phenolic acid	(Wójcik-Borowska et al., 2025)	2
S11	12.78	C_15_H_18_O_5_	279.1242	201.0914、191.1073、173.0965	-0.61	Artemargyinolide C	Terpenoids	(Sisó-Terraza et al., 2016)	2
S12	14.00	C_26_H_28_O_14_	565.1557	379.0306、295.0577	0.42	Schaftoside	Flavonoids	(Li et al., 2022)	2
S13	14.51	C_26_H_28_O_14_	565.1541	421.1917、179.1047、118.0833	-1.98	Isoschaftoside	Flavonoids	(Li et al., 2022)	2
S14	15.29	C_10_H_10_O_4_	195.0626	183.0726、153.1274、119.0832	0.07	Trans-ferulic acid	Phenolic acid	(Ramos et al., 2023)	2
S15	15.86	C_21_H_20_O_10_	433.1129	313.0724、311.2054、193.1571	-0.88	Vitexin*	Flavonoids	(Zhu et al., 2015)	2
S16	16.06	C_27_H_30_O_16_	611.1613	303.0471、300.1074、163.0393	-2.15	Rutin*	Flavonoids	(Zhu et al., 2015; Pașca et al., 2021)	2
S17	17.07	C_15_H_10_O_7_	303.0497	259.0960、165.0911、155.0024、151.1167	-0.75	Quercetin*	Flavonoids	(Zhu et al., 2015; Pașca et al., 2021)	2
S18	19.05	C_25_H_24_O_12_	517.1362	499.1242、355.1056、173.0394、135.0445	0.62	3,4-Dicaffeoylquinic acid	Phenolic acid	(Hernández et al., 2021; Rodrigues et al., 2025)	2
S19	19.30	C_25_H_24_O_12_	517.1371	500.1275 、499.1246、163.0391	-2.51	Cynarin	Phenolic acid	(Serrano et al., 2021)	2
S20	20.17	C_25_H_24_O_12_	517.1344	499.1259、177.0802、173.0653、163.0396、135.0422	0.38	3,5-Dicaffeoylquinic acid*	Phenolic acid	(Hernández et al., 2021)	2
S21	20.85	C_21_H_18_O_11_	447.0935	269.0446、151.0122、119.0835	-0.66	Apigenin-7-O-glucronide*	Flavonoids	(El-Newary et al., 2023)	2
S22	21.51	C_27_H_30_O_15_	595.1628	363.1998、287.1261、119.0862	-0.91	Datiscin*	Flavonoids	(Gifford et al., 2018)	2
S23	22.36	C_25_H_24_O_12_	517.1346	417.1524、355.1036、191.0712、175.1477、135.0378	0.77	4,5-Dicaffeoylquinic acid*	Phenolic acid	(Hernández et al., 2021)	2
S24	23.23	C_9_H_6_O_3_	163.0359	145.0276、135.0427、119.0880、102.0117、91.0026	0.89	7-Hydroxycoumarin*	Coumarins	(Luo et al., 2021)	2
S25	23.97	C_26_H_26_O_12_	531.1481	243.1024、163.0378、119.0884	-0.65	3-caffeoyl-4-feruoyl-quinic acid	Phenolic acid	(Zhang et al., 2023)	2
S26	24.22	C_26_H_26_O_12_	531.1484	335.1277、163.0362、137.0962、102.0147	-0.82	3-caffeoyl-5-feruoyl-quinic acid	Phenolic acid	(Zhang et al., 2023)	2
S27	27.52	C_15_H_10_O_6_	287.051	151.1165、133.1362、119.0874、102.0125	0.31	Luteolin*	Flavonoids	(El-Fadaly et al., 2023)	2
S28	30.06	C_15_H_10_O_6_	287.0539	213.0525、157.0635、118.0856、105.0342	-0.76	Kaempferol*	Flavonoids	(Hu et al., 2020; El-Fadaly et al., 2023)	2
S29	30.33	C_16_H_12_O_7_	317.068	287.0559、227.1265、163.1136、102.0145	0.73	6-Methoxyluteolin*	Flavonoids	(Slimestad et al., 2022)	2
S30	31.06	C_17_H_14_O_8_	347.0731	317.0647、243.1029、119.0847、102.0148	0.72	5,7,4′,5′-Tetrahydroxy-6,3′- Dimethoxyflavone*	Flavonoids	(Kamel et al., 2023)	2
S31	31.54	C_28_H_32_O_14_	593.1836	287.2058、151.0987、119.0854	-0.73	Buddleoside	Flavonoids	(Jiao et al., 2018)	2
S32	32.44	C_17_H_14_O_8_	347.0764	241.0876、135.1343、109.0874、102.0158、95.0874	-0.89	Limocitrin	Flavonoids	(Darnal et al., 2023)	2
IS	33.12	C_11_H_6_O_3_	187.0409	132.0511、131.0573、115.0549	-1.13	Psoralen	Flavonoids	(Cui et al., 2021)	2
S33	35.72	C_15_H_10_O_5_	271.0629	163.0759、119.0861、105.0351	0.78	Apigenin	Flavonoids	(Haile et al., 2021)	2
S34	36.61	C_16_H_12_O_6_	301.0728	271.0699、135.1151、119.0858	0.74	3-methoxyapigenin	Flavonoids	(Lee et al., 2018)	2
S35	37.46	C_16_H_12_O_7_	317.0651	165.0543、139.0356、119.0847	-1.69	Rhamnetin*	Flavonoids	(Ali et al., 2025)	2
S36	37.53	C_16_H_12_O_6_	301.0747	181.1221、163.0747、124.0869、119.0825	2.04	Hispidulin	Flavonoids	(Bojilov et al., 2023)	2
S37	37.96	C_17_H_14_O_7_	331.0839	163.0781、118.0857	2.25	Jaceosidin*	Flavonoids	(Fu et al., 2020)	2
S38	38.51	C_18_H_16_O_8_	361.0965	331.0871、301.0759、163.0791、105.0347	1.47	Centaureidin*	Flavonoids	(Li et al., 2022)	2
S39	39.81	C_18_H_16_O_8_	361.0937	331.0888、119.0874	0.87	Centaureidin isomer A*	Flavonoids	(Zhu et al., 2015)	3
S40	47.52	C_17_H_14_O_6_	315.0885	282.0514、136.0136、118.0863	1.69	Cirsimaritin	Flavonoids	(Basit et al., 2022)	2
S41	48.65	C_18_H_16_O_7_	345.0952	330.0217、243.0198	2.57	Eupatilin	Flavonoids	(Zhang et al., 2024)	2
S42	50.52	C_19_H_18_O_8_	375.1075	147.0917、118.0846	1.77	Casticin	Flavonoids	(Chang et al., 2021)	2
S43	51.18	C_16_H_12_O_5_	285.0793	279.0932、153.0839、118.0851	-1.29	Acacetin	Flavonoids	(Gao et al., 2022)	2
S44	53.01	C_19_H_18_O_7_	359.1133	289.1811、118.0869	0.72	5-Hydroxy-3',4',6,7-tetramethoxyflavone	Flavonoids	(Nasseri et al., 2022)	2

*VIP > 1.

Annotation confidence levels were assigned according to Sumner et al. (2007). Level 1: confirmed identification using an authentic standard and at least two independent and orthogonal properties obtained under the same experimental conditions. Level 2: putatively annotated compound based on spectral-library, accurate-mass, fragment-ion, database, and/or literature matching without confirmation using an authentic standard. Level 3: putatively characterized compound class based on class-specific spectral or physicochemical properties. Level 4: unknown or unclassified compound.

### 3.2 Multivariate statistical analysis reveals metabolic variation during AAL development

#### 3.2.1 Cluster analysis

**Figure 2:**
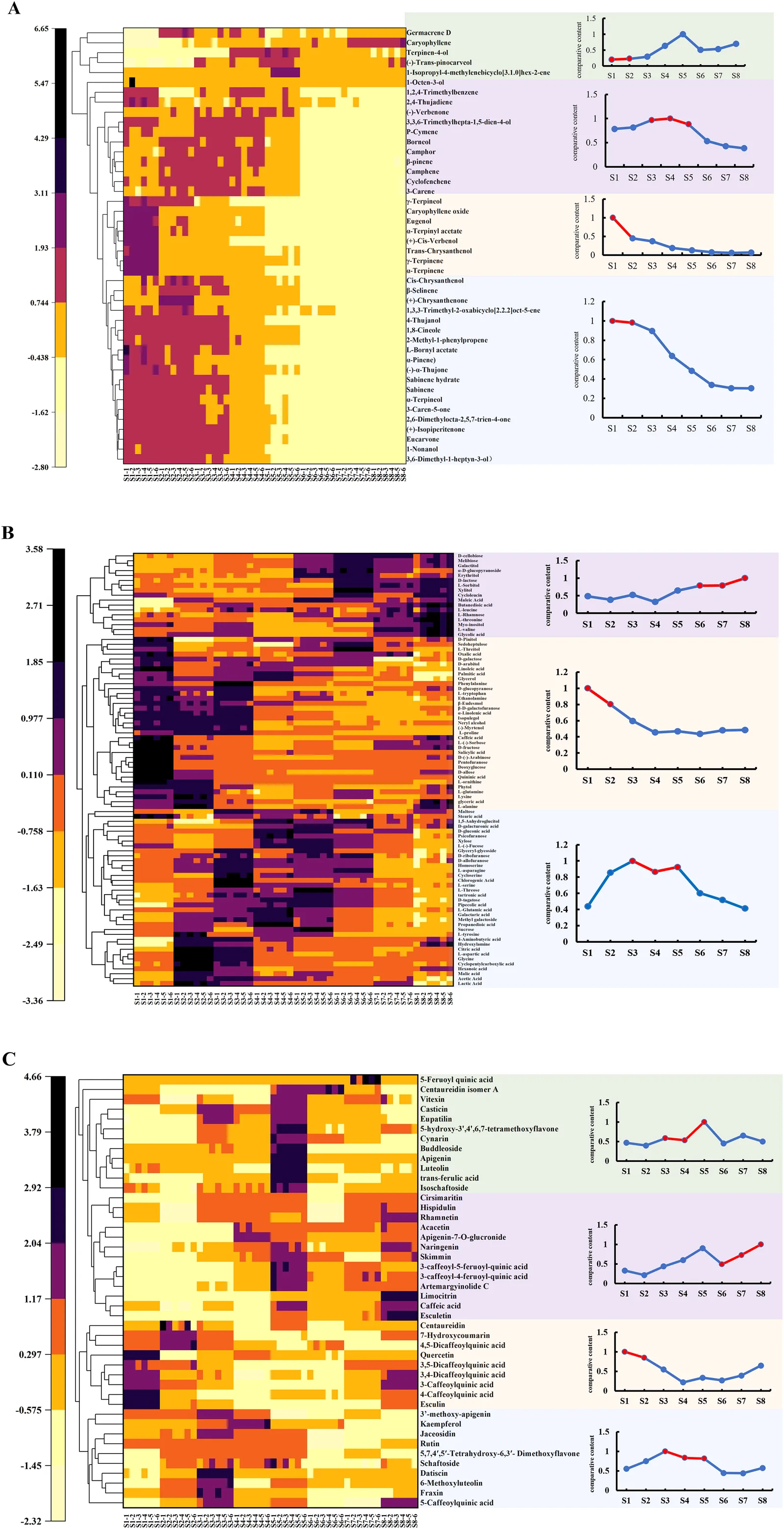
Hierarchical clustering reveals distinct metabolic profiles of AAL at different growth stages. **(A)** Volatile components detected by HS-GC-MS; **(B)** primary metabolites detected by pre-column derivatization GC-MS; **(C)** secondary metabolites detected by UHPLC-Q/TOF-MS in positive-ion mode. S1-S2 denote early growth phase, S3-S5 vigorous growth phase, and S6-S8 senescence phase.

To further explore the dynamic patterns in chemical components of AAL across different growth stages, hierarchical cluster analysis (HCA) was performed on all annotated metabolites (**Figure 2**). The HCA results are shown in the form of heat maps, which facilitate intuitive recognition of the dynamic patterns of the annotated metabolites in AAL across the growth stages. The annotated metabolites with similar dynamic patterns during AAL development were grouped together.

As shown in **Figure 2A**, the relative contents of 27 out of 44 volatile compounds were higher in the initial growth stage then decreased in the following growth stages (**Figure 2A**). In contrast, the relative contents of 11 volatile compounds such as camphor, camphene, pinene were accumulated in the early developing stages, reached the highest amount during the vigorous growing stage, and decreased afterwards. The left 5 volatile compounds showed distinct changing patterns. The contents of α-terpinen, γ-terpinene and caryophyllene oxide were in higher contents at early development stages than in vigorous and senescence stages with only their peaking time slightly different. In a former study, these metabolites showed peak accumulation around 18^th^ of June in 2018 which was the Dragon Boat Festival of that year (Chang et al., 2020), while they were in highest content around 25^th^ of April in the present study. Considering same cultivars of AAL were used as before, the climate differences between these two years might be main contributor to the difference in content peaking time. Caryophyllene was the only volatile component that accumulated continuously during the whole developing stages of AAL and showed the highest content in the senescent leaves. Caryophyllene has shown antioxidant and anti-senescence properties (Wen et al., 2022; Nodola et al., 2024). The accumulation of caryophyllene was supposed to help AAL cope with oxidative damage and other abiotic stresses during the senescence process.

The variation trends of primary metabolites analyzed by pre-column derivatization coupled with GC-MS could be summarized into three dynamic patterns (**Figure 2B**). The relative contents of 14 carbohydrate derivatives, 7 amino acids, 5 organic acids, and 10 other primary metabolites were high in the initial growth stage, then decreased in the following growing stages. In contrast, the relative contents of 14 carbohydrate derivatives, 10 amino acids, 8 organic acids, and 3 other primary metabolites showed the highest contents in the vigorous growth stage. The relative contents of 10 carbohydrate derivatives, 4 amino acids, and 3 organic acids were accumulated abundantly and peaked in AAL at the senescence stage. The higher relative abundance of D-ribofuranose in AAL at the vigorous growth stage could be attributed to the favorable temperature and sufficient sunlight during this period, which create optimal conditions for plant metabolic activities. Vigorous cell division and growth greatly increase the demand for nucleic acids and energy metabolites. As an essential constituent of RNA and ATP, the content of D-ribofuranose significantly elevated with the activation of metabolic pathways, to meet the needs of genetic information transmission and energy supply (Wu et al., 2023).

Regarding the cluster heatmap of 44 secondary metabolites analyzed by LC-MS (**Figure 2C**), the relative contents of 5 phenolic acids, 2 flavonoids and 2 coumarins showed higher relative contents at the early growth stage and exhibited a declining trend in the subsequent growth stages. In contrast, the relative contents of 22 flavonoids, 6 phenolic acids, 2 coumarins, and one terpenoid initiated accumulation at the early developmental stage, peaked at the vigorous growth stage, and then decreased gradually thereafter. 4 metabolites, namely rhamnetin, limocitrin, 5-feruloylquinic acid, and esculetin, accumulated continuously throughout the whole developmental process of AAL, with their relative contents attaining the highest level at the senescence stage. Notably, 22 out of the 26 detected flavonoids and over half of the annotated phenolic acids accumulated dramatically during early developmental stage and reached their maximum relative abundance at the vigorous growth stage. This dynamic changing pattern of these flavonoids and phenolic acids was consistent with former study of total contents of flavonoids and phenolic acids (Xue et al., 2019). This phenomenon was supposed to be closely related to the enhanced photosynthesis and metabolic activities of AAL at this stage, which supply sufficient precursors and energy for the biosynthesis of secondary metabolites (Nikolić et al., 2024). Furthermore, high light intensity and strong ultraviolet radiation were reported to trigger the activation of flavonoid biosynthetic pathways, thereby accelerate the massive accumulation of flavonoids (Zeng et al., 2020).

#### 3.2.2 OPLS-DA analysis

**Figure 3:**
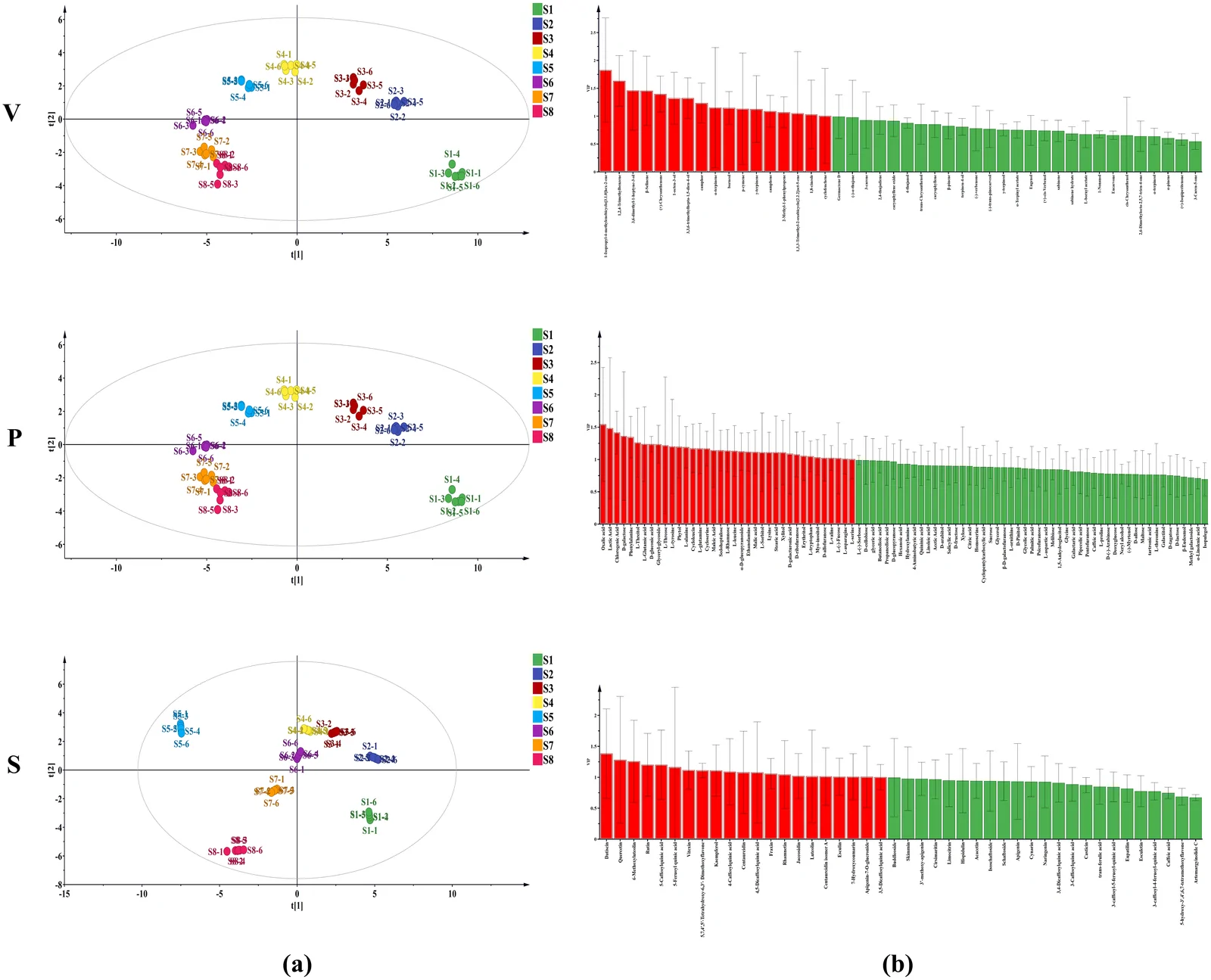
OPLS-DA scatter plots **(a)** and VIP score plots **(b)** of AAL samples for screening differential volatile metabolites (V), non-volatile primary metabolites (P) and secondary metabolites (S).

In this study, AAL samples from 8 distinct growth stages were subjected to comparative analysis using orthogonal partial least squares-discriminant analysis (OPLS-DA). As illustrated in **Figure 3**, AAL samples from the 8 growth stages were clearly segregated into 8 discrete clusters, confirming significant differences in metabolite contents across different growth stages. The model was validated via 200 permutation tests, with results summarized in **Table 4**. The R²X, R²Y, and Q² values were 0.912, 0.960, and 0.948 for HS-GC-MS; 0.979, 0.990, and 0.986 for pre-column derivatization GC-MS; and 0.972, 0.987, and 0.983 for UHPLC-Q/TOF-MS, respectively. The Q² intercepts from the 200-permutation tests were -0.518, -0.644, and -0.645, respectively, supporting the absence of overfitting. Differential chemical components, including 17 volatile components, 37 non-volatile primary metabolites, and 21 non-volatile secondary metabolites, were selected using the criterion VIP > 1 (**Table 4**). The corresponding 200-permutation test plots are provided in **Supplementary Figure 5**.

**Table 4:** OPLS-DA results of AAL samples at different growth stages based on HS-GC–MS, pre-column derivatization GC-MS and UHPLC-Q/TOF-MS.

Metabolite	Model verification results			Permutation results		Number of characteristic
	*R*2*X* (cum)	*R*2*Y* (cum)	*Q*2 (cum)	*R*2	*Q*2	Peaks with VIP > 1
V	0.912	0.960	0.948	0.719	−0.518	17
P	0.979	0.990	0.986	0.0511	-0.644	37
S	0.972	0.987	0.983	0.0635	-0.645	21

Of the 17 differential volatile components identified, 11 compounds, including β-selinene, α-terpinene, 1,8-cineole, 3,6-dimethyl-1-heptyne-3-ol, γ-terpinene, 1,3,3-trimethyl-2-oxabicyclo[2.2.2]oct-5-ene, and chrysanthenone (8 monoterpenes, 2 alcohols, 1 sesquiterpene), exhibited significantly higher relative abundances at the early growth stage than at the vigorous growth and senescence stages (**Supplementary Figure 2**, **Supplementary Table 2**). In contrast, the other 6 metabolites (4 monoterpenes and 2 aromatic hydrocarbons), namely camphor, camphene, p-cymene, borneol, 1,2,4-trimethylbenzene, and 1-isopropyl-4-methylenebicyclo[3.1.0]hex-2-ene, showed markedly higher relative abundances at the vigorous growth stage compared with the other two growth stages (**Supplementary Figure 2**, **Supplementary Table 2**).

Among the 37 differential primary metabolites, 12 compounds, including lysine, L-glutamine, D-galactose, phytol, L-tyrosine, oxalic acid, stearic acid, and ethanolamine (5 amino acids, 3 sugar derivatives, 1 organic acid, 3 other metabolites) had significantly higher relative abundances at the early growth stage relative to the vigorous growth and senescence stages. By contrast, 15 metabolites, including L-threose, L-fucose, D-ribofuranose, L-serine, cycloserine, D-allofuranose, L-asparagine, D-gluconic acid, malic acid, and D-galacturonic acid (7 sugar derivatives, 5 amino acids, 3 organic acids), displayed elevated relative abundances at the vigorous growth stage relative to the other two growth stages. The remaining 10 metabolites, including L-valine, maleic acid, xylitol, L-sorbitol, L-rhamnose, erythritol, myo-inositol, and α-D-glucopyranoside (6 sugar derivatives, 3 amino acids, 1 organic acid), accumulated continuously and peaked in relative abundance at the senescence stage (**Supplementary Figure 3**, **Supplementary Table 3**).

Of the 21 differential secondary metabolites identified, 7 compounds, including 4-caffeoylquinic acid, 4,5-dicaffeoylquinic acid, 3,5-dicaffeoylquinic acid, esculin, quercetin, 7-hydroxycoumarin, and centaureidin (3 phenolic acids, 2 flavonoids, 2 coumarins), exhibited significantly higher relative abundances at the early growth stage than at the vigorous growth and senescence stages. By contrast, 12 metabolites, including apigenin-7-O-glucuronide, datiscin, kaempferol, jaceosidin, 5-caffeoylquinic acid, 6-methoxyluteolin, vitexin, luteolin, rutin, and 5,7,4′,5′-tetrahydroxy-6,3′-dimethoxyflavone (11 flavonoids, 1 phenolic acid), showed significantly higher relative abundances at the vigorous growth stage than at the other two growth stages. The remaining 2 metabolites, namely rhamnetin and 5-feruloylquinic acid (1 phenolic acid, 1 flavonoid), accumulated substantially and reached the maximum relative abundance at the senescence stage (**Supplementary Figure 4**, **Supplementary Table 4**).

### 3.3 Elucidation of regulatory mechanisms for AAL constituents based on KEGG pathway analysis

**Figure 4:**
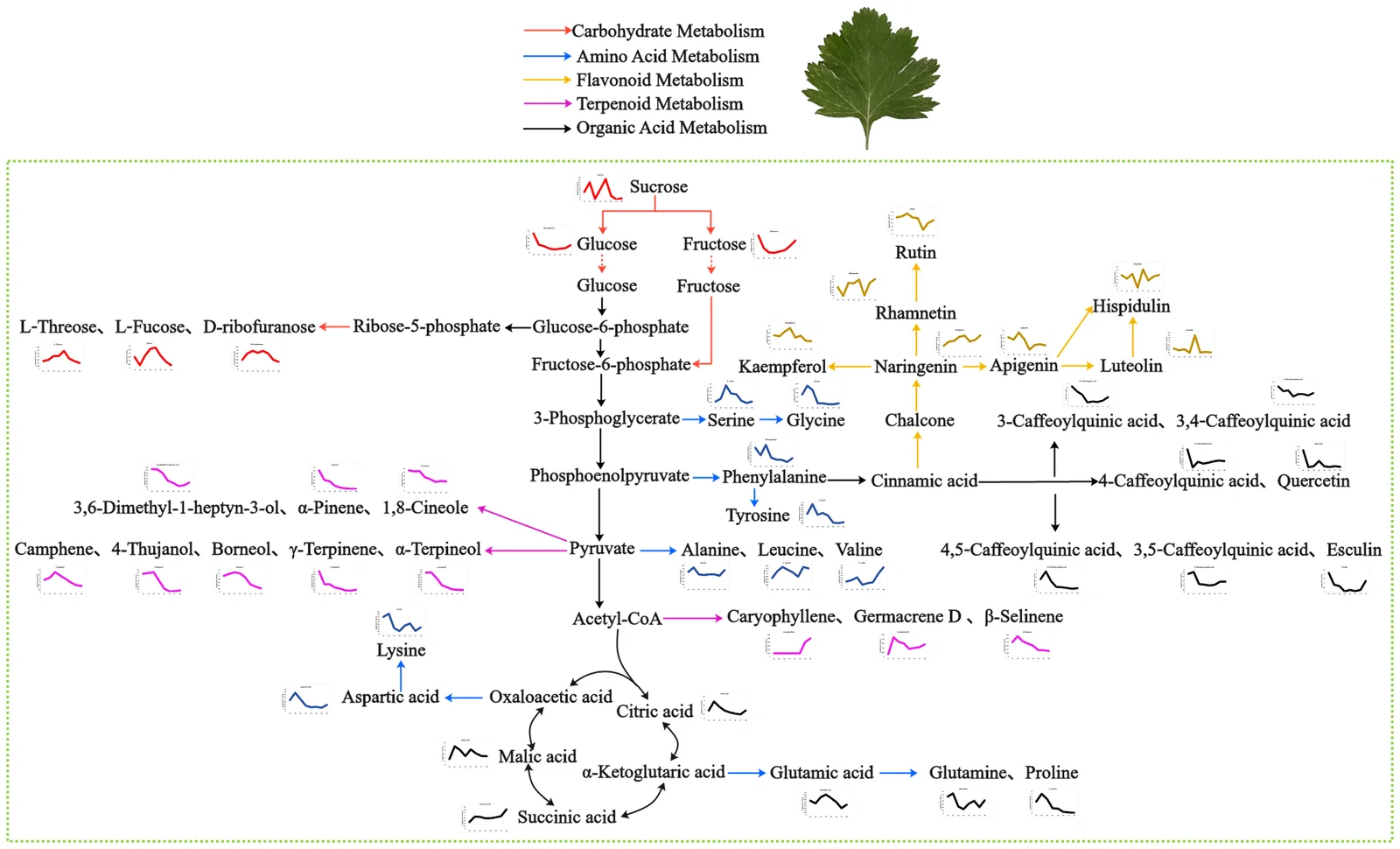
Integration of dynamic changing patterns of main constituents with their proposed biosynthetic pathways information for elucidation of metabolic regulation mechanism of AAL during development.

To further explore the metabolic relationships in AAL across different growth stages, the detected metabolites were imported into the KEGG metabolic pathway database for mapping. Consequently, the metabolic pathways of major compounds in AAL at different growth stages were integrated (**Figure 4**), based on which the dynamic variation patterns of AAL metabolites were further investigated. **Figure 4** presents a proposed pathway framework based on KEGG annotations and previously reported Artemisia pathways (Lee et al., 2023; He et al., 2024). Additionally, the dynamic changing curves of annotated metabolites were also integrated in this framework. As illustrated in **Figure 4**, the metabolic network of AAL throughout growth stages mainly involves five core pathways: carbohydrate metabolism, amino acid metabolism, flavonoid metabolism, terpenoid metabolism, and organic acid metabolism. Sucrose, the energy source for plant activities, is catabolized via the glycolysis pathway to generate key intermediates, such as glucose-6-phosphate, 3-phosphoglycerate, pyruvic acid, and acetyl-CoA. These key intermediates are essential precursors for energy production by involving in tricarboxylic acid cycle (TCA cycle) and biosynthesis of primary and secondary metabolites such as amino acids, flavonoids and terpenoids.

Plants are more susceptible to environmental and biotic stresses (e.g., low temperature and pathogen infection) at the early growth stages, which might promote the extensive accumulation of metabolites with antibacterial activity to defend against pathogenic invasion (He et al., 2024). Specifically, metabolites derived from the phenylpropanoid pathway significantly accumulated during AAL development. Through enzymatic transformation of critical intermediates (e.g., cinnamic acid and caffeic acid), this pathway facilitated the extensive synthesis of antibacterial phenolic acids, such as caffeoylquinic acids. In addition, the monoterpene biosynthesis pathway produced substantial amounts of volatile monoterpenes (e.g., 1,8-cineole and α-pinene) via the MEP pathway. These compounds were reported to possess excellent antibacterial and insect-resistant activities (Gizaw et al., 2025). Extensive accumulation of these bioactive metabolites at the early growth stage might enable AAL to effectively defend against diseases, insect pests, and adverse environmental stresses.

At the vigorous growth stage, the photosynthetic efficiency of AAL is significantly enhanced, with substantial accumulation of photosynthates (Nikolić et al., 2024). Core intermediates of carbohydrate metabolism are preferentially diverted to the biosynthesis of secondary metabolites. Besides the sustained biosynthesis of phenolic acids through the phenylpropanoid pathway, some carbon skeletons are catalyzed by enzymes such as chalcone synthase and flavone synthase, leading to the massive synthesis of flavonoids, such as kaempferol, luteolin, rutin, naringenin, and apigenin (Zeng et al., 2020).

At the senescence stage, the overall metabolic activity of AAL declines progressively (Jha et al., 2023), resulting in reduced contents of most metabolites, whereas metabolites with potent antioxidant activity accumulate significantly (Kim, 2013; Ames-Sibin et al., 2018; Bozkurt et al., 2023; Ali et al., 2024). Specifically, for most primary and secondary metabolites, the synthesis rate is lower than the degradation rate, leading to a significant reduction in their relative abundances. Intermediates of TCA cycle intermediates is adaptively regulated. Amino acids are gradually catabolized and fed into the TCA cycle for energy production, facilitating the recycling and reuse of nutrients. Meanwhile, the contents of some flavonoids derived from the phenylpropanoid pathway and caryophyllene that synthesized via the sesquiterpene biosynthesis pathway were enhanced during the senescence stage. The accumulation of metabolites such as rhamnetin and caryophyllene with antioxidant activity (Kim, 2013; Ames-Sibin et al., 2018; Bozkurt et al., 2023; Ali et al., 2024) might help AAL in mitigating oxidative damage stresses.

## 4 Conclusions

In this study, mass spectrometry-based metabolomic platforms were applied for comprehensive analysis of chemical components of AAL during development. In total, 44 volatile and 132 non-volatile metabolites were annotated and relatively quantified. Multivariate statistical analyses showed significant differences in metabolite composition across growing phases. Further, a proposed metabolic regulatory network encompassing five core pathways: carbohydrate metabolism, amino acid metabolism, flavonoid metabolism, terpenoid metabolism, and organic acid metabolism, was constructed to present an overview of the dynamic changes of central metabolites in AAL during development. Dynamic metabolic profiling revealed that AAL was preferentially allocated to the synthesis of defensive compounds at the early growth stage, leading to prominent accumulation of phenolic acids and monoterpenoids. At the vigorous growth stage, AAL exhibited enhanced photosynthetic capacity and metabolic efficiency; carbon skeletons were largely channeled into flavonoid biosynthesis, causing a marked enrichment of flavonoids such as rutin. At the senescence stage, AAL showed a substantial decline in overall metabolic activity. Antioxidant metabolites exemplified by caryophyllene, sugar derivatives, flavonoids and caffeic acid derivatives accumulated abundantly might contribute to mitigate oxidative damage and stress associated with leaf senescence. This study preliminarily elucidated the dynamic accumulation profiles of chemical constituents in AAL, which would provide fundamental data for the optimal harvest-time determination and quality evaluation of AAL according to application purpose and target products.

## Data Availability

The original contributions presented in the study are included in the article/**Supplementary Material**. Further inquiries can be directed to the corresponding authors.
