# Supplementary material for: Exploring dynamic changes in metabolic profiles of Artemisia argyi leaves across different growth stages via plant metabolomics
Source: Front Plant Sci. 2026 Aug 11;17:1913814. doi: 10.3389/fpls.2026.1913814 (PMC13503534; doi:10.3389/fpls.2026.1913814)
Supplement: Supplementary file 1 [file DataSheet1.docx]

Supplementary Material

# 1 Supplementary Figures

**Supplementary Figure S1.** Morphology of AAL samples harvested at different growth stages. S1-S2 denote early growth, S3-S5 vigorous growth, and S6-S8 senescence.

**Supplementary Figure S2.** Relative contents of differential volatile components in AAL during development.

**Supplementary Figure S3.** Relative contents of differential primary metabolites in AAL during development.

**Supplementary Figure S4.** Relative contents of differential secondary metabolites in AAL during development.

**A HS-GC-MS**

**B Pre-column derivatization GC-MS**

**C UHPLC-Q/TOF-MS**

**Supplementary Figure S5.** Results of the 200-permutation tests for the OPLS-DA models based on (A) HS-GC-MS, (B) pre-column derivatization GC-MS, and (C) UHPLC-Q/TOF-MS data. The Q² intercepts were -0.518, -0.644, and -0.645, respectively.

**Supplementary Tables**

**Supplementary Table S1.** Sample information of *Artemisia argyi* leaves.

| Sample | Collection date | Growth stage | Specimen number | Morphological descriptor |
| --- | --- | --- | --- | --- |
| S1 | 2024.04.25 | early growth stage | 20240425AALS1 | Young, small, deeply dissected leaf |
| S2 | 2024.05.20 |  | 20240520AALS2 | Expanding leaf with increased blade area |
| S3 | 2024.06.10 | vigorous growth stage | 20240610AALS3 | Fully expanded mature green leaf |
| S4 | 2024.07.05 |  | 20240705AALS4 | Fully expanded broad green leaf |
| S5 | 2024.08.01 |  | 20240801AALS5 | Mature leaf at maximum apparent expansion |
| S6 | 2024.08.30 | senescence stage | 20240830AALS6 | Onset of senescence with early discoloration |
| S7 | 2024.09.25 |  | 20240925AALS7 | Progressive senescence with visible chlorosis |
| S8 | 2024.10.21 |  | 20241021AALS8 | Advanced senescence with marked chlorosis and necrotic lesions |

**Supplementary Table S2. Relative abundance of volatile components in AAL at different growth stages**

| No. | Compounds | Relative abundance at different growth stages (%) (mean±SD) | | | | | | | |
| --- | --- | --- | --- | --- | --- | --- | --- | --- | --- |
|  |  | S1 | S2 | S3 | S4 | S5 | S6 | S7 | S8 |
| V1 | 3,6-dimethyl-1-heptyne-3-ol* | 0.74±0.05^A^ | 0.73±0.03^A^ | 0.60±0.03^B^ | 0.32±0.16^C^ | 0.24±0.12^CD^ | 0.13±0.06^D^ | 0.15±0.01^D^ | 0.25±0.01^CD^ |
| V2 | 1-Nonanol | 0.09±0.01^A^ | 0.09±0.01^A^ | 0.08±0.01^B^ | 0.04±0.02^C^ | 0.01±0.01^D^ | 0.01±0.01^D^ | 0.01±0.01^D^ | 0.01±0.01^D^ |
| V3 | 3-carene | 0.31±0.04^BC^ | 0.37±0.01^B^ | 0.42±0.01^A^ | 0.32±0.16^BC^ | 0.28±0.14^C^ | 0.20±0.10^D^ | 0.18±0.01^D^ | 0.14±0.04^E^ |
| V4 | α-pinene | 0.91±0.19^A^ | 0.61±0.07^B^ | 0.52±0.02^C^ | 0.24±0.12^D^ | 0.12±0.06^E^ | 0.07±0.03^F^ | 0.05±0.01^FG^ | 0.04±0.01^G^ |
| V5 | Cyclofenchene* | 10.28±1.27^AB^ | 11.64±0.37^A^ | 12.22±0.28^A^ | 10.41±0.31^B^ | 9.19±0.75^C^ | 6.08±0.99^D^ | 5.90±0.29^D^ | 4.94±1.54^E^ |
| V6 | 1-Isopropyl-4-Methylenebicyclo[3.1.0]hex-2-ene* | 0.11±0.01^B^ | 0.05±0.01^C^ | 0.04±0.01^C^ | 0.12±0.12^B^ | 2.18±1.30^A^ | 0.01±0.01^D^ | 0.01±0.01^D^ | 0.01±0.01^D^ |
| V7 | Camphene* | 2.48±0.16^C^ | 2.73±0.17^BC^ | 4.05±0.05^A^ | 3.34±0.32^B^ | 2.66±0.14^BC^ | 1.94±0.05^D^ | 1.38±0.06^E^ | 1.24±0.08^E^ |
| V8 | 2,4-thujadiene | 0.04±0.01^A^ | 0.02±0.01^A^ | 0.02±0.01^A^ | 0.02±0.01^A^ | 0.02±0.01^A^ | 0.02±0.01^A^ | 0.02±0.01^A^ | 0.02±0.01^A^ |
| V9 | Sabinene | 2.35±0.33^B^ | 2.85±0.28^A^ | 1.92±0.11^B^ | 0.49±0.24^C^ | 0.15±0.08^D^ | 0.03±0.02^E^ | 0.04±0.01^E^ | 0.06±0.02^E^ |
| V10 | β-pinene | 0.65±0.12^BC^ | 0.89±0.03^AB^ | 0.93±0.10^A^ | 0.70±0.34^B^ | 0.54±0.27^C^ | 0.40±0.20^D^ | 0.37±0.02^D^ | 0.35±0.04^D^ |
| V11 | 1-octen-3-ol* | 1.82±0.23^A^ | 1.71±0.03^A^ | 1.62±0.09^A^ | 1.39±0.29^B^ | 0.71±0.55^C^ | 0.33±0.17^D^ | 0.44±0.03^D^ | 0.48±0.04^D^ |
| V12 | 1,3,3-Trimethyl-2-Oxabicyclo[2.2.2]oct-5-ene* | 0.27±0.03^B^ | 0.31±0.02^A^ | 0.23±0.01^C^ | 0.15±0.07^D^ | 0.11±0.05^D^ | 0.10±0.07^D^ | 0.08±0.02^E^ | 0.06±0.01^E^ |
| V13 | 1,2,4-Trimethylbenzene* | 0.14±0.01^AB^ | 0.01±0.01^C^ | 0.11±0.01^BC^ | 0.17±0.11^A^ | 0.08±0.09^BC^ | 0.01±0.01^C^ | 0.05±0.01^C^ | 0.04±0.01^C^ |
| V14 | 2-Methyl-1-phenylpropene* | 0.09±0.01^A^ | 0.08±0.01^A^ | 0.10±0.01^A^ | 0.08±0.04^A^ | 0.04±0.02^B^ | 0.02±0.01^C^ | 0.02±0.01^C^ | 0.02±0.01^C^ |
| V15 | α-terpinene* | 0.70±0.04^A^ | 0.21±0.02^B^ | 0.25±0.01^B^ | 0.12±0.06^C^ | 0.05±0.03^D^ | 0.02±0.01^E^ | 0.02±0.01^E^ | 0.03±0.01^E^ |
| V16 | P-cymene* | 4.73±0.21^C^ | 4.27±0.15^D^ | 5.43±0.20^B^ | 7.15±1.55^A^ | 4.98±0.35^C^ | 2.39±0.21^E^ | 2.15±0.13^E^ | 1.99±0.09^F^ |
| V17 | 1,8-cineole* | 27.73±1.85^A^ | 26.45±0.89^A^ | 26.87±0.42^A^ | 18.45±9.05^B^ | 15.83±7.79^B^ | 12.02±5.90^C^ | 13.30±0.36^C^ | 12.79±0.36^C^ |
| V18 | γ-terpinene* | 0.94±0.08^A^ | 0.35±0.03^B^ | 0.35±0.02^B^ | 0.20±0.10^C^ | 0.15±0.07^C^ | 0.07±0.04^D^ | 0.06±0.01^D^ | 0.10±0.02^D^ |
| V19 | Sabinene hydrate | 5.74±0.52^B^ | 6.33±0.24^A^ | 4.71±0.19^C^ | 1.36±0.67^D^ | 0.24±0.12^E^ | 0.11±0.18^E^ | 0.07±0.03^E^ | 0.12±0.02^E^ |
| V20 | 3,3,6-trimethylhepta-1,5-dien-4-ol* | 0.38±0.02^AB^ | 0.30±0.01^B^ | 0.40±0.02^A^ | 0.39±0.16^AB^ | 0.30±0.13^B^ | 0.09±0.04^C^ | 0.07±0.01^C^ | 0.07±0.01^C^ |
| V21 | 4-thujanol | 1.32±0.09^A^ | 1.38±0.04^A^ | 1.43±0.02^A^ | 0.82±0.40^B^ | 0.24±0.12^C^ | 0.04±0.02^D^ | 0.06±0.02^D^ | 0.11±0.02^CD^ |
| V22 | (-)-α-thujone | 0.34±0.04^A^ | 0.30±0.03^AB^ | 0.25±0.04^BC^ | 0.16±0.08^CD^ | 0.13±0.06^D^ | 0.09±0.05^D^ | 0.10±0.01^D^ | 0.10±0.01^D^ |
| V23 | (+)-Chrysanthenone* | 0.28±0.02^B^ | 0.52±0.02^A^ | 0.27±0.02^B^ | 0.14±0.07^C^ | 0.17±0.08^C^ | 0.05±0.03^D^ | 0.02±0.01^D^ | 0.04±0.01^D^ |
| V24 | Cis-Chrysanthenol | 0.11±0.04^A^ | 0.13±0.04^A^ | 0.11±0.02^A^ | 0.06±0.03^B^ | 0.04±0.02^B^ | 0.03±0.01^B^ | 0.03±0.01^B^ | 0.03±0.01^B^ |
| V25 | Eucarvone | 20.02±1.41^A^ | 19.24±0.73^A^ | 17.19±0.38^B^ | 10.28±5.05^C^ | 7.48±3.72^D^ | 4.73±2.32^E^ | 5.01±0.21^E^ | 5.26±0.21^E^ |
| V26 | (-)-trans-pinocarveol | 0.05±0.02^B^ | 0.05±0.02^B^ | 0.15±0.01^A^ | 0.14±0.08^A^ | 0.14±0.07^A^ | 0.10±0.05^AB^ | 0.12±0.02^A^ | 0.11±0.02^AB^ |
| V27 | Camphor* | 1.23±0.12^C^ | 1.71±0.11^B^ | 2.43±0.12^A^ | 1.95±0.12^B^ | 1.97±0.13^B^ | 0.92±0.04^D^ | 0.63±0.04^E^ | 0.62±0.06^E^ |
| V28 | Trans-Chrysanthenol | 3.26±0.36^A^ | 1.51±0.11^B^ | 1.28±0.06^B^ | 0.60±0.30^C^ | 0.43±0.25^CD^ | 0.30±0.15^D^ | 0.28±0.01^D^ | 0.30±0.02^D^ |
| V29 | Borneol* | 1.03±0.06^BC^ | 1.15±0.09^AB^ | 1.25±0.07^A^ | 1.19±0.44^AB^ | 0.95±0.36^C^ | 0.49±0.21^D^ | 0.34±0.02^E^ | 0.23±0.05^F^ |
| V30 | Terpinen-4-ol | 0.45±0.02^C^ | 0.29±0.01^D^ | 0.46±0.02^C^ | 0.68±0.33^B^ | 0.78±0.39^A^ | 0.56±0.28^BC^ | 0.52±0.01^C^ | 0.49±0.02^C^ |
| V31 | α-terpineol | 0.79±0.09^A^ | 0.80±0.06^A^ | 0.57±0.03^B^ | 0.28±0.14^C^ | 0.19±0.09^D^ | 0.11±0.05^E^ | 0.09±0.01^E^ | 0.08±0.01^E^ |
| V32 | (-)-verbenone | 0.05±0.01^A^ | 0.05±0.01^A^ | 0.05±0.01^A^ | 0.05±0.02^A^ | 0.05±0.02^A^ | 0.03±0.01^B^ | 0.03±0.01^B^ | 0.03±0.01^B^ |
| V33 | γ-terpineol | 0.28±0.05^A^ | 0.25±0.02^A^ | 0.10±0.05^B^ | 0.01±0.01^C^ | 0.02±0.01^C^ | 0.01±0.01^C^ | 0.01±0.01^C^ | 0.01±0.01^C^ |
| V34 | 2,6-Dimethylocta-2,5,7-trien-4-one | 0.14±0.01^A^ | 0.12±0.01^A^ | 0.10±0.01^A^ | 0.06±0.03^B^ | 0.03±0.02^C^ | 0.01±0.01^D^ | 0.01±0.01^D^ | 0.01±0.01^D^ |
| V35 | (+)-cis-Verbenol | 0.89±0.10^A^ | 0.36±0.01^B^ | 0.28±0.02^C^ | 0.04±0.02^D^ | 0.02±0.01^D^ | 0.01±0.01^D^ | 0.01±0.01^D^ | 0.01±0.01^D^ |
| V36 | (+)-Isopiperitenone | 0.19±0.02^A^ | 0.18±0.01^A^ | 0.16±0.01^A^ | 0.07±0.04^B^ | 0.04±0.02^C^ | 0.02±0.01^D^ | 0.02±0.01^D^ | 0.02±0.01^D^ |
| V37 | L-bornyl acetate | 0.16±0.04^A^ | 0.10±0.01^B^ | 0.10±0.01^B^ | 0.03±0.01^C^ | 0.01±0.01^D^ | 0.01±0.01^D^ | 0.01±0.01^D^ | 0.01±0.01^D^ |
| V38 | α-Terpinyl acetate | 0.19±0.01^A^ | 0.10±0.01^B^ | 0.07±0.01^C^ | 0.03±0.01^D^ | 0.02±0.01^E^ | 0.01±0.01^E^ | 0.01±0.01^E^ | 0.01±0.01^E^ |
| V39 | Eugenol | 0.22±0.04^A^ | 0.11±0.01^B^ | 0.07±0.02^C^ | 0.03±0.02^D^ | 0.01±0.01^E^ | 0.01±0.01^E^ | 0.01±0.01^E^ | 0.01±0.01^E^ |
| V40 | 3-Caren-5-one | 3.15±0.27^A^ | 2.89±0.21^A^ | 2.42±0.12^B^ | 1.23±0.63^C^ | 0.89±0.45^D^ | 0.45±0.22^E^ | 0.46±0.10^E^ | 0.47±0.05^E^ |
| V41 | Caryophyllene | 0.07±0.01^E^ | 0.19±0.01^D^ | 0.21±0.01^C^ | 0.23±0.11^B^ | 0.18±0.09^D^ | 0.22±0.11^BC^ | 0.32±0.02^A^ | 0.37±0.05^A^ |
| V42 | Germacrene D | 0.03±0.01^F^ | 0.22±0.02^A^ | 0.17±0.01^B^ | 0.15±0.07^C^ | 0.09±0.05^E^ | 0.10±0.05^DE^ | 0.11±0.01^D^ | 0.14±0.02^CD^ |
| V43 | β-Selinene* | 0.20±0.01^B^ | 0.28±0.01^A^ | 0.21±0.01^B^ | 0.18±0.07^C^ | 0.15±0.01^D^ | 0.09±0.05^E^ | 0.09±0.01^E^ | 0.08±0.01^E^ |
| V44 | Caryophyllene oxide | 0.06±0.01^A^ | 0.04±0.01^B^ | 0.02±0.01^C^ | 0.01±0.01^C^ | 0.01±0.01^C^ | 0.01±0.01^C^ | 0.01±0.01^C^ | 0.01±0.01^C^ |

**Note *: VIP > 1. Different uppercase letters indicate significant differences among growth stages for the same compound (*P* < 0.05); identical letters indicate no significant difference.**

**Supplementary Table S3. Relative abundance of primary metabolites in AAL at different growth stages**

| No. | Compounds | Relative abundance at different growth stages (%) (mean±SD) | | | | | | | |
| --- | --- | --- | --- | --- | --- | --- | --- | --- | --- |
|  |  | S1 | S2 | S3 | S4 | S5 | S6 | S7 | S8 |
| P1 | Lactic Acid, 2TMS* | 0.18±0.05^BC^ | 0.23±0.02^A^ | 0.19±0.02^AB^ | 0.17±0.01^C^ | 0.17±0.01^C^ | 0.17±0.01^C^ | 0.18±0.07^BC^ | 0.12±0.01^D^ |
| P2 | Hexanoic acid, TMS | 0.02±0.03^C^ | 0.05±0.02^A^ | 0.02±0.01^C^ | 0.01±0.01^D^ | 0.01±0.01^D^ | 0.01±0.01^D^ | 0.01±0.01^D^ | 0.03±0.01^B^ |
| P3 | Glycolic acid, 2TMS | 0.02±0.01^B^ | 0.02±0.01^B^ | 0.02±0.01^B^ | 0.01±0.01^C^ | 0.01±0.01^C^ | 0.01±0.01^C^ | 0.02±0.01^B^ | 0.03±0.01^A^ |
| P4 | L-alanine，2TMS* | 0.25±0.04^C^ | 0.32±0.05^A^ | 0.21±0.01^DE^ | 0.20±0.01^E^ | 0.21±0.01^DE^ | 0.21±0.02^DE^ | 0.19±0.10^F^ | 0.28±0.03^B^ |
| P5 | Hydroxylamine, 3TMS | 0.11±0.03^D^ | 0.15±0.01^A^ | 0.13±0.01^B^ | 0.12±0.01^C^ | 0.12±0.01^C^ | 0.12±0.01^C^ | 0.10±0.05^E^ | 0.15±0.02^A^ |
| P6 | Oxalic acid，2TMS* | 0.38±0.06^A^ | 0.29±0.03^DE^ | 0.27±0.02^F^ | 0.29±0.01^DE^ | 0.30±0.03^D^ | 0.33±0.01^C^ | 0.34±0.15^B^ | 0.28±0.03^EF^ |
| P7 | L-proline，TMS | 4.35±0.66^C^ | 7.16±0.16^A^ | 5.29±2.82^B^ | 1.95±0.36^D^ | 1.79±0.98^E^ | 0.62±0.13^F^ | 0.43±0.39^G^ | 0.24±0.06^H^ |
| P8 | Cyclopentylcarboxylic acid, TMS | 0.32±0.24^C^ | 0.58±0.12^A^ | 0.41±0.13^B^ | 0.23±0.03^D^ | 0.18±0.04^E^ | 0.15±0.03^H^ | 0.16±0.10^G^ | 0.17±0.03^F^ |
| P9 | (-)-Myrtenol，TMS | 0.16±0.02^A^ | 0.14±0.02^B^ | 0.09±0.07^C^ | 0.01±0.01^E^ | 0.03±0.01^D^ | 0.01±0.01^E^ | 0.01±0.01^E^ | 0.01±0.01^E^ |
| P10 | Propanedioic acid, 2TMS | 0.14±0.02^D^ | 0.15±0.01^C^ | 0.18±0.06^B^ | 0.22±0.06^A^ | 0.15±0.01^C^ | 0.11±0.03^E^ | 0.06±0.04^F^ | 0.06±0.01^F^ |
| P11 | Neryl alcohol，TMS | 0.14±0.02^B^ | 0.16±0.02^A^ | 0.10±0.07^C^ | 0.01±0.01^D^ | 0.01±0.01^D^ | 0.01±0.01^D^ | 0.01±0.01^D^ | 0.01±0.01^D^ |
| P12 | L-leucine，2TMS* | 0.07±0.02^G^ | 0.12±0.02^D^ | 0.15±0.03^A^ | 0.13±0.03^C^ | 0.11±0.03^E^ | 0.08±0.03^F^ | 0.15±0.07^A^ | 0.14±0.02^B^ |
| P13 | L-valine，2TMS* | 0.25±0.01^E^ | 0.31±0.10^D^ | 0.42±0.18^C^ | 0.12±0.03^G^ | 0.18±0.03^F^ | 0.22±0.16^E^ | 0.56±0.23^B^ | 0.83±0.06^A^ |
| P14 | Isopulegol，2TMS | 0.09±0.01^A^ | 0.08±0.01^B^ | 0.07±0.01^C^ | 0.04±0.01^D^ | 0.03±0.01^E^ | 0.02±0.01^F^ | 0.02±0.01^F^ | 0.02±0.01^F^ |
| P15 | L-serine，2TMS* | 0.06±0.03^F^ | 0.10±0.09^D^ | 0.28±0.07^A^ | 0.17±0.01^B^ | 0.16±0.05^C^ | 0.07±0.02^E^ | 0.04±0.02^G^ | 0.06±0.01^F^ |
| P16 | Pipecolic acid, TMS | 0.13±0.12^E^ | 0.29±0.01^C^ | 0.30±0.02^B^ | 0.38±0.03^A^ | 0.38±0.04^A^ | 0.27±0.08^D^ | 0.12±0.08^F^ | 0.13±0.02^E^ |
| P17 | Ethanolamine, 3TMS* | 0.39±0.04^A^ | 0.36±0.03^AB^ | 0.34±0.06^B^ | 0.28±0.03^CD^ | 0.29±0.04^C^ | 0.23±0.02^E^ | 0.17±0.09^F^ | 0.27±0.03^D^ |
| P18 | Glycerol, 3TMS | 2.34±0.21^B^ | 2.17±0.20^C^ | 2.50±0.11^A^ | 2.35±0.57^B^ | 1.51±0.16^D^ | 1.32±0.02^E^ | 0.88±0.58^F^ | 0.77±0.07^G^ |
| P19 | L-ornithine，3TMS | 9.28±0.89^A^ | 7.01±2.00^B^ | 3.93±0.78^C^ | 2.98±0.04^D^ | 2.97±0.05^D^ | 3.12±0.37^C^ | 2.49±1.33^E^ | 2.69±0.23^DE^ |
| P20 | Maleic Acid，2TMS* | 0.01±0.06^BC^ | 0.01±0.02^BC^ | 0.01±0.01^BC^ | 0.01±0.04^BC^ | 0.02±0.02^A^ | 0.01±0.01^BC^ | 0.01±0.05^BC^ | 0.01±0.07^BC^ |
| P21 | [Succinic Acid](https://www.chembk.com/cn/chem/Succinic%20Acid), 2TMS | 0.50±0.27^G^ | 0.89±0.06^D^ | 0.88±0.13^DE^ | 0.77±0.04^F^ | 0.78±0.06^F^ | 0.84±0.21^E^ | 0.93±0.46^C^ | 1.42±0.13^A^ |
| P22 | β-Eudesmol, TMS | 0.07±0.01^A^ | 0.05±0.01^B^ | 0.05±0.01^B^ | 0.04±0.01^C^ | 0.03±0.01^D^ | 0.02±0.01^E^ | 0.02±0.01^E^ | 0.02±0.01^E^ |
| P23 | Glyceric acid，3TMS | 0.39±0.03^AB^ | 0.39±0.05^AB^ | 0.32±0.03^C^ | 0.28±0.03^D^ | 0.24±0.01^E^ | 0.25±0.05^E^ | 0.25±0.14^E^ | 0.48±0.05^A^ |
| P24 | Acetic Acid，2TMS | 0.09±0.08^DE^ | 0.18±0.01^A^ | 0.16±0.01^B^ | 0.14±0.01^C^ | 0.11±0.01^D^ | 0.09±0.03^DE^ | 0.06±0.05^F^ | 0.02±0.01^G^ |
| P25 | Cycloleucin, 2TMS* | 0.44±0.05^D^ | 0.30±0.08^F^ | 0.21±0.01^G^ | 0.27±0.09^FG^ | 0.52±0.21^C^ | 0.66±0.20^B^ | 0.42±0.24^E^ | 0.76±0.07^A^ |
| P26 | L-threonine，3TMS | 0.07±0.01^EF^ | 0.07±0.01^EF^ | 0.06±0.01^F^ | 0.07±0.03^EF^ | 0.13±0.04^E^ | 0.21±0.04^D^ | 0.24±0.13^C^ | 0.43±0.04^A^ |
| P27 | Glycine，3TMS | 0.13±0.14^C^ | 0.29±0.04^A^ | 0.18±0.10^B^ | 0.03±0.01^CD^ | 0.03±0.01^CD^ | 0.02±0.01^D^ | 0.02±0.01^D^ | 0.03±0.01^CD^ |
| P28 | Malic acid，3TMS* | 3.16±0.10^H^ | 10.13±0.33^A^ | 8.10±0.02^B^ | 4.76±2.03^F^ | 8.45±0.11^B^ | 6.24±0.71^C^ | 4.84±0.90^E^ | 4.73±0.42^G^ |
| P29 | Salicylic acid，2TMS | 0.13±0.07^A^ | 0.04±0.01^E^ | 0.05±0.01^D^ | 0.07±0.01^B^ | 0.06±0.01^C^ | 0.05±0.01^D^ | 0.05±0.03^D^ | 0.05±0.01^D^ |
| P30 | L-Threitol，4TMS* | 0.15±0.05^B^ | 0.07±0.02^D^ | 0.09±0.01^CD^ | 0.07±0.02^D^ | 0.11±0.04^C^ | 0.19±0.07^A^ | 0.06±0.03^E^ | 0.05±0.01^F^ |
| P31 | L-aspartic acid，3TMS | 0.94±0.59^C^ | 1.54±0.20^A^ | 1.02±0.39^B^ | 0.49±0.12^D^ | 0.36±0.04^F^ | 0.39±0.04^E^ | 0.32±0.17^G^ | 0.56±0.06^D^ |
| P32 | 4-Aminobutyric acid，3TMS | 0.65±0.97^H^ | 1.89±0.02^A^ | 1.66±0.37^B^ | 1.16±0.09^C^ | 0.96±0.14^D^ | 0.79±0.02^E^ | 0.75±0.39^F^ | 1.31±0.12^G^ |
| P33 | L-Threose，4TMS* | 0.13±0.01^DE^ | 0.15±0.03^D^ | 0.21±0.02^B^ | 0.21±0.04^B^ | 0.29±0.06^A^ | 0.17±0.02^C^ | 0.13±0.03^DE^ | 0.10±0.01^E^ |
| P34 | L-(-)-Fucose，5TMS* | 0.15±0.06^D^ | 0.04±0.01^F^ | 0.17±0.03^C^ | 0.26±0.22^B^ | 0.28±0.05^A^ | 0.17±0.05^C^ | 0.09±0.02^E^ | 0.04±0.01^F^ |
| IS | 2-Isopropylmalic acid，3TMS | 4.08±0.07^AB^ | 4.03±0.04^C^ | 4.10±0.08^A^ | 4.11±0.31^A^ | 4.09±0.06^A^ | 4.11±0.12^A^ | 4.03±0.53^C^ | 4.05±0.19^B^ |
| P35 | Xylose，4TMS | 0.51±0.19^D^ | 0.37±0.16^E^ | 0.72±0.20^C^ | 1.12±0.10^A^ | 1.13±0.13^A^ | 0.82±0.23^B^ | 0.35±0.21^F^ | 0.36±0.03^F^ |
| P36 | Cycloserine，3TMS* | 0.05±0.03^E^ | 0.02±0.10^H^ | 0.22±0.06^A^ | 0.14±0.02^B^ | 0.13±0.01^C^ | 0.12±0.06^D^ | 0.03±0.01^G^ | 0.04±0.01^F^ |
| P37 | L-Glutamic acid, 3TMS* | 0.32±0.03^D^ | 0.26±0.07^E^ | 0.40±0.03^B^ | 0.46±0.05^A^ | 0.38±0.05^C^ | 0.29±0.07^E^ | 0.15±0.08^G^ | 0.23±0.03^F^ |
| P38 | Phenylalanine, 2TMS* | 0.55±0.15^B^ | 0.22±0.32^E^ | 0.88±0.27^A^ | 0.31±0.09^C^ | 0.22±0.05^E^ | 0.21±0.11^E^ | 0.11±0.10^F^ | 0.28±0.04^D^ |
| P39 | L-asparagine，3TMS* | 0.07±0.40^F^ | 1.51±0.21^B^ | 1.80±0.43^A^ | 0.96±0.21^C^ | 1.46±0.19^B^ | 1.72±0.78^A^ | 0.03±0.11^G^ | 0.03±0.01^G^ |
| P40 | Homoserine，3TMS | 1.54±0.48^E^ | 2.47±0.51^B^ | 2.86±0.57^A^ | 2.19±0.08^C^ | 2.20±0.08^C^ | 1.68±1.00^D^ | 0.26±0.18^F^ | 0.24±0.03^F^ |
| P41 | D-arabitol，5TMS | 0.44±0.06^A^ | 0.38±0.02^C^ | 0.42±0.03^B^ | 0.42±0.02^B^ | 0.43±0.01^A^ | 0.38±0.08^C^ | 0.22±0.11^D^ | 0.27±0.02^D^ |
| P42 | Xylitol，5TMS* | 0.16±0.02^F^ | 0.12±0.03^G^ | 0.14±0.03^F^ | 0.17±0.02^E^ | 0.32±0.20^D^ | 0.50±0.13^A^ | 0.30±0.15^C^ | 0.46±0.05^B^ |
| P43 | Lysine，4TMS* | 0.70±0.11^B^ | 0.83±0.28^A^ | 0.24±0.11^E^ | 0.12±0.09^G^ | 0.34±0.07^C^ | 0.43±0.16^D^ | 0.13±0.07^F^ | 0.27±0.04^E^ |
| P44 | Erythritol，4TMS* | 0.34±0.06^F^ | 0.34±0.25^F^ | 0.40±0.21^E^ | 0.45±1.07^D^ | 2.22±0.13^A^ | 1.99±0.66^B^ | 0.50±0.41^C^ | 1.18±0.11^B^ |
| P45 | L-glutamine，3TMS* | 1.08±0.15^AB^ | 1.28±0.36^A^ | 0.45±0.20^FG^ | 0.28±0.27^H^ | 0.63±0.09^EF^ | 0.81±0.21^CD^ | 0.41±0.23^G^ | 0.84±0.10^BC^ |
| P46 | Psicofuranose，4TMS | 0.18±0.03^D^ | 0.15±0.02^E^ | 0.21±0.05^C^ | 0.28±0.03^A^ | 0.25±0.01^B^ | 0.22±0.04^CD^ | 0.12±0.07^F^ | 0.15±0.02^E^ |
| P47 | Citric acid，4TMS | 0.91±0.94^C^ | 1.93±0.31^A^ | 1.34±0.28^B^ | 0.91±0.14^C^ | 0.69±0.07^D^ | 0.55±0.07^E^ | 0.47±0.26^F^ | 0.91±0.09^C^ |
| P48 | Methyl galactoside，4TMS | 0.13±0.03^D^ | 0.18±0.02^AB^ | 0.20±0.01^A^ | 0.20±0.03^A^ | 0.17±0.02^B^ | 0.14±0.02^CD^ | 0.08±0.05^E^ | 0.09±0.01^E^ |
| P49 | D-Pinitol，5TMS | 18.62±3.51^A^ | 14.58±0.89^DE^ | 16.50±1.08^CD^ | 17.87±0.27^BC^ | 18.04±0.54^AB^ | 18.09±1.06^AB^ | 13.63±6.73^E^ | 18.38±1.55^A^ |
| P50 | D-fructose，5TMS | 3.84±1.35^A^ | 1.68±0.60^C^ | 0.80±0.17^F^ | 0.73±0.36^G^ | 0.99±0.30^E^ | 1.25±1.02^D^ | 2.13±1.06^B^ | 3.20±0.29^A^ |
| P51 | D-galactose，5TMS* | 0.60±0.10^A^ | 0.44±0.02^CD^ | 0.45±0.06^C^ | 0.37±0.12^DE^ | 0.48±0.02^BC^ | 0.50±0.25^B^ | 0.08±0.11^F^ | 0.31±0.03^E^ |
| P52 | L-(-)-Sorbose，5TMS | 2.92±1.03^A^ | 1.31±0.42^C^ | 0.67±0.16^F^ | 0.53±0.33^G^ | 0.78±0.25^E^ | 1.04±0.89^D^ | 1.68±0.87^B^ | 2.21±0.20^B^ |
| P53 | D-allose，5TMS | 2.92±1.03^A^ | 1.96±0.82^B^ | 0.78±0.19^D^ | 0.47±0.05^F^ | 0.41±0.07^G^ | 0.67±0.55^E^ | 1.41±0.80^C^ | 2.76±0.26^A^ |
| P54 | L-tyrosine，3TMS* | 0.15±0.25^D^ | 0.47±0.20^A^ | 0.13±0.09^DE^ | 0.27±0.03^B^ | 0.21±0.12^C^ | 0.05±0.01^F^ | 0.04±0.02^G^ | 0.06±0.01^F^ |
| P55 | L-Sorbitol，6TMS* | 0.14±0.03^D^ | 0.12±0.04^DE^ | 0.14±0.01^D^ | 0.10±0.05^E^ | 0.04±0.47^F^ | 0.89±0.09^B^ | 0.78±0.13^C^ | 1.01±0.32^A^ |
| P56 | Tartronic acid，3TMS | 0.08±0.03^F^ | 0.18±0.07^D^ | 0.24±0.01^A^ | 0.23±0.02^B^ | 0.24±0.02^A^ | 0.19±0.04^C^ | 0.10±0.06^E^ | 0.10±0.01^E^ |
| P57 | β-D-galactofuranose，5TMS | 1.51±0.03^A^ | 1.59±0.20^A^ | 1.55±0.44^A^ | 0.79±0.30^B^ | 0.40±0.03^D^ | 0.43±0.01^C^ | 0.42±0.23^C^ | 0.81±0.08^B^ |
| P58 | D-tagatose，6TMS | 0.54±1.08^F^ | 0.75±0.12^E^ | 0.93±0.05^C^ | 1.01±0.01^A^ | 0.99±0.01^B^ | 0.86±0.19^D^ | 0.42±0.26^G^ | 0.40±0.04^G^ |
| P59 | D-gluconic acid，6TMS* | 0.85±0.15^E^ | 0.93±0.16^D^ | 1.25±0.81^C^ | 2.23±0.21^A^ | 1.66±0.45^B^ | 1.18±0.16^C^ | 0.73±0.74^F^ | 0.12±0.01^G^ |
| P60 | Palmitic acid，TMS | 0.62±0.10^BC^ | 0.54±0.07^CD^ | 0.68±0.05^B^ | 0.71±0.09^A^ | 0.56±0.06^CD^ | 0.46±0.04^D^ | 0.28±0.18^E^ | 0.27±0.02^E^ |
| P61 | Galactaric acid，6TMS | 0.15±0.07^E^ | 0.25±0.02^C^ | 0.31±0.05^B^ | 0.34±0.07^A^ | 0.23±0.01^CD^ | 0.18±0.05^DE^ | 0.08±0.05^F^ | 0.07±0.01^G^ |
| P62 | D-allofuranose，5TMS* | 1.82±0.04^CD^ | 1.92±0.13^BC^ | 2.05±0.09^A^ | 1.94±0.06^B^ | 1.87±0.01^C^ | 1.75±0.19^D^ | 1.21±0.61^E^ | 1.75±0.16^D^ |
| P63 | D-ribofuranose，4TMS* | 0.15±0.03^DE^ | 0.25±0.02^C^ | 0.29±0.03^A^ | 0.26±0.02^BC^ | 0.29±0.03^A^ | 0.25±0.06^C^ | 0.15±0.03^DE^ | 0.12±0.02^F^ |
| P64 | Deoxyglucose，4TMS | 1.05±0.45^A^ | 0.44±0.04^B^ | 0.36±0.09^C^ | 0.23±0.05^DE^ | 0.18±0.01^F^ | 0.22±0.05^E^ | 0.25±0.13^D^ | 0.41±0.04^B^ |
| P65 | Myo-inositol，6TMS* | 3.08±0.78^BC^ | 2.31±0.49^C^ | 2.98±1.13^BC^ | 0.08±0.28^G^ | 0.13±0.33^F^ | 0.08±1.17^G^ | 3.14±1.41^B^ | 4.21±0.43^A^ |
| P66 | Caffeic acid，3TMS | 0.72±0.21^A^ | 0.46±0.04^C^ | 0.37±0.11^D^ | 0.25±0.02^F^ | 0.31±0.03^E^ | 0.39±0.06^D^ | 0.42±0.22^C^ | 0.74±0.07^A^ |
| P67 | Phytol，TMS* | 0.51±0.01^A^ | 0.48±0.06^AB^ | 0.38±0.05^C^ | 0.33±0.04^D^ | 0.37±0.04^C^ | 0.31±0.02^D^ | 0.27±0.14^E^ | 0.46±0.05^B^ |
| P68 | Linoleic acid, TMS | 0.37±0.12^A^ | 0.25±0.06^C^ | 0.32±0.01^B^ | 0.29±0.04^BC^ | 0.19±0.06^D^ | 0.14±0.02^E^ | 0.12±0.07^E^ | 0.14±0.02^E^ |
| P69 | α-Linolenic acid, TMS | 0.65±0.05^A^ | 0.59±0.03^B^ | 0.54±0.17^C^ | 0.29±0.06^D^ | 0.16±0.08^F^ | 0.10±0.03^G^ | 0.12±0.06^G^ | 0.18±0.02^E^ |
| P70 | L-Rhamnose，4TMS* | 0.27±0.08^E^ | 0.19±0.07^F^ | 0.28±0.08^E^ | 0.12±0.11^G^ | 0.34±0.07^D^ | 0.25±0.09^E^ | 0.41±0.18^C^ | 0.57±0.05^A^ |
| P71 | L-tryptophan，3TMS* | 0.50±0.10^B^ | 0.40±0.16^C^ | 0.58±0.20^A^ | 0.25±0.08^E^ | 0.28±0.10^DE^ | 0.20±0.09^F^ | 0.16±0.08^G^ | 0.24±0.04^E^ |
| P72 | Stearic acid，TMS* | 0.15±0.04^A^ | 0.07±0.04^D^ | 0.14±0.01^A^ | 0.13±0.01^B^ | 0.11±0.03^C^ | 0.07±0.03^D^ | 0.11±0.05^C^ | 0.11±0.01^C^ |
| P73 | α-D-glucopyranoside，4TMS* | 0.09±0.02^F^ | 0.12±0.02^E^ | 0.13±0.01^D^ | 0.10±0.06^F^ | 0.19±0.02^C^ | 0.22±0.03^A^ | 0.17±0.07^B^ | 0.13±0.01^D^ |
| P74 | Glyceryl-glycoside，6TMS* | 0.31±0.01^D^ | 0.38±0.10^C^ | 0.51±0.12^A^ | 0.35±0.10^CD^ | 0.47±0.08^B^ | 0.29±0.03^D^ | 0.25±0.11^E^ | 0.17±0.02^F^ |
| P75 | Galactitol，6TMS | 0.49±0.02^G^ | 0.53±0.11^F^ | 0.73±0.09^E^ | 0.98±0.17^D^ | 1.25±0.07^A^ | 1.25±0.14^A^ | 0.85±0.43^C^ | 1.23±0.12^B^ |
| P76 | D-galacturonic acid，5TMS* | 0.28±0.05^E^ | 0.22±0.01^F^ | 0.22±0.05^F^ | 0.37±0.03^C^ | 0.40±0.03^A^ | 0.36±0.04^B^ | 0.30±0.13^D^ | 0.33±0.09^D^ |
| P77 | D-cellobiose，8TMS | 0.17±0.05^F^ | 0.15±0.06^F^ | 0.20±0.05^E^ | 0.23±0.16^E^ | 0.46±0.03^C^ | 0.50±0.01^A^ | 0.38±0.20^D^ | 0.48±0.05^B^ |
| P78 | Sucrose，8TMS | 6.32±0.07^D^ | 14.21±0.08^B^ | 0.73±0.01^F^ | 8.52±0.05^C^ | 16.95±1.50^A^ | 3.31±0.02^E^ | 0.23±0.01^G^ | 1.13±0.03^F^ |
| P79 | Maltose，8TMS | 0.35±0.03^E^ | 0.20±0.01^G^ | 0.71±0.01^A^ | 0.59±0.02^C^ | 0.67±0.13^B^ | 0.14±0.01^H^ | 0.24±0.02^F^ | 0.46±0.03^D^ |
| P80 | D-lactose，8TMS | 0.27±0.10^F^ | 0.16±0.03^G^ | 0.25±0.07^F^ | 0.48±0.11^E^ | 0.74±0.25^C^ | 1.01±0.07^B^ | 0.76±0.38^C^ | 1.15±0.11^A^ |
| P81 | Melibiose，8TMS | 0.31±0.02^G^ | 0.37±0.14^F^ | 0.58±0.05^E^ | 0.82±0.26^D^ | 1.22±0.08^A^ | 1.20±0.21^AB^ | 0.80±0.41^C^ | 1.33±0.14^A^ |
| P82 | Chlorogenic Acid，6TMS* | 0.07±0.01^F^ | 0.54±0.71^B^ | 1.47±0.62^A^ | 0.29±0.02^E^ | 0.35±0.05^D^ | 0.38±0.04^C^ | 0.19±0.20^E^ | 0.01±0.01^G^ |
| P83 | Quininic acid，6TMS | 4.95±1.69^A^ | 1.89±1.36^B^ | 0.09±0.07^F^ | 0.12±0.18^F^ | 0.24±0.18^E^ | 0.29±0.44^E^ | 0.46±0.50^C^ | 0.01±0.01^G^ |
| P84 | D-glucopyranose，5TMS | 0.33±0.04^A^ | 0.32±0.07^A^ | 0.29±0.20^B^ | 0.03±0.01^F^ | 0.03±0.01^F^ | 0.07±0.05^E^ | 0.13±0.07^D^ | 0.23±0.03^C^ |
| P85 | D-(-)-Arabinose，6TMS | 0.84±0.36^A^ | 0.35±0.05^C^ | 0.23±0.11^D^ | 0.08±0.01^G^ | 0.11±0.06^F^ | 0.18±0.03^E^ | 0.20±0.11^DE^ | 0.39±0.07^B^ |
| P86 | Pentofuranose，4TMS | 1.60±0.87^A^ | 0.40±0.11^B^ | 0.22±0.11^C^ | 0.08±0.01^E^ | 0.09±0.03^E^ | 0.15±0.02^D^ | 0.15±0.08^D^ | 0.23±0.04^C^ |
| P87 | 1,5-Anhydroglucitol，4TMS | 0.15±0.05^F^ | 0.13±0.06^F^ | 0.29±0.10^C^ | 0.40±0.07^A^ | 0.35±0.04^B^ | 0.35±0.07^B^ | 0.22±0.12^D^ | 0.28±0.03^C^ |
| P88 | Sedoheptulose，4TMS* | 0.46±0.19^A^ | 0.15±0.05^E^ | 0.23±0.12^D^ | 0.03±0.02^F^ | 0.28±0.06^B^ | 0.39±0.10^A^ | 0.26±0.12^C^ | 0.23±0.02^D^ |

**Note *: VIP > 1. Different uppercase letters indicate significant differences among growth stages for the same compound (*P* < 0.05); identical letters indicate no significant difference.**

**Supplementary Table S4. Relative abundance of secondary metabolites in AAL at different growth stages**

| No. | Compounds | Relative abundance at different growth stages (%) (mean±SD) | | | | | | | |
| --- | --- | --- | --- | --- | --- | --- | --- | --- | --- |
|  |  | S1 | S2 | S3 | S4 | S5 | S6 | S7 | S8 |
| S1 | 5-Caffeoylquinic acid* | 1.75±0.03^D^ | 2.00±0.02^C^ | 3.71±0.18^A^ | 2.33±0.03^B^ | 2.99±0.01^B^ | 1.67±0.02^D^ | 0.73±0.03^E^ | 3.74±0.08^A^ |
| S2 | Esculin* | 2.16±0.01^A^ | 0.83±0.07^C^ | 0.67±0.01^C^ | 0.07±0.01^D^ | 0.24±0.01^D^ | 0.10±0.01^D^ | 0.24±0.01^D^ | 1.25±0.03^B^ |
| S3 | Fraxin* | 2.20±0.01^D^ | 3.42±0.01^B^ | 4.15±0.10^A^ | 2.75±0.09^C^ | 2.93±0.06^C^ | 1.88±0.04^E^ | 1.49±0.08^F^ | 2.01±0.03^DE^ |
| S4 | Esculetin | 0.31±0.03^E^ | 0.16±0.01^G^ | 0.01±0.01^H^ | 0.22±0.01^F^ | 2.69±0.16^B^ | 0.70±0.01^D^ | 1.69±0.04^C^ | 4.36±0.05^A^ |
| S5 | Skimmin | 0.28±0.01^G^ | 0.48±0.01^F^ | 0.58±0.04^E^ | 0.77±0.06^D^ | 1.11±0.04^A^ | 0.78±0.01^D^ | 0.36±0.02^FG^ | 0.98±0.03^B^ |
| S6 | 3-Caffeoylquinic acid | 10.50±0.08^B^ | 7.68±0.20^C^ | 5.86±0.14^D^ | 1.51±0.13^F^ | 1.98±0.05^E^ | 1.77±0.01^F^ | 2.52±0.12^D^ | 4.45±0.46^A^ |
| S7 | 4-Caffeoylquinic acid* | 1.73±0.01^A^ | 0.10±0.61^C^ | 0.61±0.01^B^ | 0.44±0.01^B^ | 0.53±0.03^B^ | 0.65±0.02^B^ | 0.72±0.03^B^ | 0.77±0.04^B^ |
| S8 | Caffeic acid | 3.10±0.05^D^ | 1.76±0.10^F^ | 2.80±0.10^E^ | 2.46±0.05^E^ | 4.69±0.12^B^ | 3.15±0.19^D^ | 3.58±0.08^C^ | 5.11±0.01^A^ |
| S9 | Naringenin | 0.74±0.01^G^ | 1.07±0.01^F^ | 1.14±0.10^E^ | 1.47±0.03^C^ | 1.56±0.04^B^ | 1.09±0.07^F^ | 1.21±0.02^D^ | 1.58±0.02^A^ |
| S10 | 5-Feruoyl quinic acid* | 1.58±0.28^C^ | 0.56±0.02^E^ | 0.50±0.01^E^ | 0.63±0.08^DE^ | 1.24±0.12^D^ | 0.21±0.01^F^ | 20.49±7.27^A^ | 1.41±0.03^C^ |
| S11 | Artemargyinolide C | 1.05±0.23^G^ | 0.85±0.12^H^ | 1.85±0.07^F^ | 2.03±0.12^E^ | 5.45±0.18^A^ | 2.66±0.05^D^ | 3.73±0.06^B^ | 3.73±0.15^B^ |
| S12 | Schaftoside | 6.13±0.27^E^ | 9.02±0.45^B^ | 9.56±0.98^A^ | 9.15±0.63^B^ | 9.33±2.58^AB^ | 6.67±0.05^D^ | 5.56±0.79^F^ | 7.31±0.60^C^ |
| S13 | Isoschaftoside | 6.83±0.57^B^ | 3.68±0.27^F^ | 6.55±0.22^BC^ | 6.63±0.41^BC^ | 9.62±0.27^A^ | 3.64±0.13^F^ | 5.72±0.02^D^ | 5.95±0.25^CD^ |
| S14 | trans-ferulic acid | 1.53±0.09^F^ | 1.51±0.04^F^ | 1.84±0.13^E^ | 3.03±0.08^C^ | 6.85±0.27^A^ | 1.73±0.08^F^ | 1.53±0.01^F^ | 3.04±0.05^C^ |
| S15 | Vitexin* | 1.96±0.02^B^ | 0.90±0.04^F^ | 1.65±0.08^C^ | 1.13±0.03^E^ | 2.15±0.06^A^ | 1.52±0.10^D^ | 1.82±0.01^BC^ | 1.48±0.33^D^ |
| S16 | Rutin* | 0.90±0.01^B^ | 0.96±0.01^B^ | 1.11±0.01^A^ | 0.89±0.01^B^ | 0.86±0.01^B^ | 0.22±0.01^D^ | 0.60±0.01^C^ | 0.74±0.02^C^ |
| S17 | Quercetin* | 1.16±0.09^A^ | 0.09±0.01^D^ | 0.15±0.01^BC^ | 0.48±0.02^B^ | 0.14±0.01^C^ | 0.10±0.01^D^ | 0.15±0.01^BC^ | 0.11±0.01^D^ |
| S18 | 3,4-Dicaffeoylquinic acid | 1.95±0.02^A^ | 1.40±0.01^CD^ | 1.49±0.08^BC^ | 0.76±0.07^F^ | 1.26±0.05^D^ | 1.04±0.13^E^ | 0.89±0.03^CD^ | 1.17±0.03^B^ |
| S19 | Cynarin | 0.41±0.01^F^ | 0.55±0.01^E^ | 1.25±0.14^C^ | 0.77±0.01^D^ | 1.86±0.06^A^ | 1.32±0.03^B^ | 0.99±0.01^D^ | 0.65±0.02^E^ |
| S20 | 3,5-Dicaffeoylquinic acid* | 4.11±0.34^B^ | 4.53±0.48^A^ | 1.80±0.07^D^ | 1.66±0.13^D^ | 1.48±0.02^E^ | 1.65±0.04^D^ | 2.35±0.16^C^ | 2.35±0.12^C^ |
| S21 | Apigenin-7-O-glucronide* | 0.50±0.14^D^ | 0.15±0.01^E^ | 0.60±0.01^C^ | 2.46±0.09^A^ | 1.93±0.03^B^ | 0.81±0.01^C^ | 1.08±0.01^C^ | 1.91±0.07^B^ |
| S22 | Datiscin* | 0.15±0.09^D^ | 0.57±0.03^B^ | 2.50±0.01^A^ | 0.51±0.02^B^ | 0.27±0.01^D^ | 0.18±0.01^D^ | 0.34±0.01^C^ | 0.28±0.01^D^ |
| S23 | 4,5-Dicaffeoylquinic acid* | 3.42±0.13^B^ | 5.54±0.25^A^ | 2.84±0.01^C^ | 1.23±0.03^D^ | 1.13±0.03^D^ | 0.95±0.14^E^ | 0.72±0.01^G^ | 0.87±0.04^F^ |
| S24 | 7-Hydroxycoumarin* | 2.43±0.01^C^ | 3.74±0.08^A^ | 2.92±0.02^B^ | 0.37±0.01^F^ | 1.01±0.09^D^ | 0.41±0.01^E^ | 1.03±0.04^D^ | 2.34±0.10^C^ |
| S25 | 3-caffeoyl-4-feruoyl-quinic acid | 0.07±0.01^F^ | 0.03±0.01^G^ | 0.18±0.01^E^ | 0.23±0.01^D^ | 0.95±0.07^A^ | 0.46±0.02^C^ | 0.74±0.03^B^ | 0.52±0.02^C^ |
| S26 | 3-caffeoyl-5-feruoyl-quinic acid | 0.03±0.01^G^ | 0.08±0.01^F^ | 0.17±0.01^E^ | 0.36±0.01^D^ | 1.28±0.10^A^ | 0.50±0.03^C^ | 0.96±0.01^B^ | 0.47±0.02^C^ |
| S27 | Luteolin* | 0.52±0.28^B^ | 0.38±0.01^C^ | 0.46±0.02^BC^ | 0.19±0.01^D^ | 1.59±0.02^A^ | 0.20±0.01^D^ | 0.25±0.01^D^ | 0.21±0.01^D^ |
| S28 | Kaempferol* | 0.67±0.01^C^ | 0.62±0.04^C^ | 0.88±0.05^B^ | 1.06±0.04^A^ | 0.55±0.02^D^ | 0.65±0.08^C^ | 0.37±0.01^E^ | 0.35±0.01^E^ |
| S29 | 6-Methoxyluteolin* | 0.23±0.01^D^ | 0.52±0.01^A^ | 0.74±0.01^A^ | 0.14±0.01^E^ | 0.49±0.01^B^ | 0.33±0.01^C^ | 0.30±0.02^C^ | 0.32±0.01^C^ |
| S30 | 5,7,4′,5′-Tetrahydroxy-6,3′-Dimethoxyflavone* | 0.14±0.01^F^ | 0.77±0.01^BC^ | 0.79±0.01^B^ | 0.84±0.01^A^ | 0.73±0.07^C^ | 0.22±0.02^E^ | 0.45±0.01^D^ | 0.06±0.01^G^ |
| S31 | Buddleoside | 0.18±0.02^C^ | 0.27±0.01^B^ | 0.30±0.01^B^ | 0.18±0.01^C^ | 0.73±0.07^A^ | 0.05±0.01^F^ | 0.09±0.01^D^ | 0.07±0.01^E^ |
| S32 | Limocitrin | 0.03±0.01^A^ | 0.03±0.01^A^ | 0.03±0.01^A^ | 0.03±0.01^A^ | 0.06±0.01^B^ | 0.06±0.01^B^ | 0.05±0.01^B^ | 0.15±0.01^C^ |
| IS | Psoralen | 0.09±0.01^AB^ | 0.09±0.01^AB^ | 0.10±0.01^A^ | 0.11±0.01^A^ | 0.10±0.07^A^ | 0.09±0.01^AB^ | 0.09±0.01^AB^ | 0.09±0.03^B^ |
| S33 | Apigenin | 0.84±0.01^B^ | 0.71±0.01^C^ | 1.10±0.01^A^ | 0.73±0.02^C^ | 0.17±0.01^G^ | 0.42±0.02^E^ | 0.46±0.01^D^ | 0.40±0.01^F^ |
| S34 | 3’-methoxy-apigenin | 0.71±0.01^D^ | 0.15±0.01^E^ | 1.11±0.03^B^ | 1.20±0.01^B^ | 0.98±0.01^C^ | 0.16±0.01^E^ | 1.08±0.05^C^ | 1.42±0.01^A^ |
| S35 | Rhamnetin* | 0.80±0.01^C^ | 0.17±0.01^E^ | 1.11±0.01^B^ | 1.08±0.01^B^ | 1.35±0.02^A^ | 0.19±0.01^D^ | 1.11±0.02^B^ | 1.39±0.04^A^ |
| S36 | Hispidulin | 0.87±0.01^C^ | 0.66±0.96^D^ | 0.95±0.07^B^ | 0.10±0.01^F^ | 1.23±0.02^A^ | 0.54±0.01^E^ | 0.79±0.02^C^ | 0.89±0.02^C^ |
| S37 | Jaceosidin* | 4.64±0.05^D^ | 5.29±0.13^C^ | 7.57±0.02^A^ | 5.87±0.13^B^ | 5.89±0.20^B^ | 1.93±0.01^G^ | 3.69±0.01^E^ | 3.16±0.04^F^ |
| S38 | Centaureidin* | 0.81±0.05^B^ | 2.25±1.12^A^ | 0.29±0.06^D^ | 1.12±0.05^B^ | 0.13±0.08^E^ | 0.16±0.30^E^ | 0.73±0.13^C^ | 0.70±0.02^C^ |
| S39 | Centaureidin isomer A* | 4.11±0.26^D^ | 3.63±0.23^E^ | 3.35±0.08^F^ | 3.66±0.23^E^ | 5.36±0.04^B^ | 5.56±0.18^A^ | 4.39±0.02^C^ | 3.70±0.12^E^ |
| S40 | Cirsimaritin | 12.74±0.37^H^ | 13.72±0.01^G^ | 18.76±0.64^B^ | 16.08±0.33^C^ | 19.54±0.36^A^ | 15.85±0.15^D^ | 15.33±0.12^E^ | 14.32±0.17^F^ |
| S41 | Eupatilin | 0.85±0.03^D^ | 0.97±0.01^C^ | 1.62±0.02^A^ | 1.39±0.03^B^ | 1.59±0.04^A^ | 0.97±0.01^C^ | 0.97±0.02^C^ | 0.69±0.01^E^ |
| S42 | Casticin | 0.09±0.01^D^ | 0.08±0.01^D^ | 0.05±0.01^E^ | 1.24±0.06^A^ | 0.97±0.01^B^ | 0.98±0.04^B^ | 0.91±0.07^C^ | 1.24±0.04^A^ |
| S43 | Acacetin | 0.35±0.01^E^ | 0.43±0.02^D^ | 0.79±0.07^B^ | 0.59±0.03^C^ | 1.18±0.02^A^ | 0.52±0.02^C^ | 0.63±0.03^C^ | 0.74±0.02^B^ |
| S44 | 5-hydroxy-3',4',6,7-tetramethoxyflavone | 0.36±0.01^E^ | 0.45±0.02^D^ | 0.72±0.07^B^ | 0.64±0.03^C^ | 0.90±0.05^A^ | 0.70±0.03^B^ | 0.66±0.08^C^ | 0.70±0.01^B^ |

**Note *: VIP > 1. Different uppercase letters indicate significant differences among growth stages for the same compound (*P* < 0.05); identical letters indicate no significant difference.**
